# Multi-layered proteogenomic analysis unravels cancer metastasis directed by MMP-2 and focal adhesion kinase signaling

**DOI:** 10.1038/s41598-021-96635-7

**Published:** 2021-08-24

**Authors:** Yumi Kwon, Seong-Jun Park, Binh Thanh Nguyen, Mi Jeong Kim, Sejin Oh, Hwanho Lee, Narae Park, Hyun Seok Kim, Min-Jung Kang, Byung Soh Min, Jin-Won Lee, Eun Gyeong Yang, Cheolju Lee

**Affiliations:** 1 Center for Theragnosis, Korea Institute of Science and Technology, 5 Hwarangro-14-gil, Seongbuk-gu, Seoul, 02792 Republic of Korea; 2 Department of Life Science and Research Institute for Natural Sciences, Hanyang University, Seoul, 04763 Korea; 3 Molecular Recognition Research Center, KIST, Seoul, 02792 Korea; 4 Division of Bio-Medical Science & Technology, KIST School, Korea University of Science and Technology, Seoul, 02792 Korea; 5 Severance Biomedical Science Institute, Yonsei University College of Medicine, Seoul, 06229 Korea; 6 Brain Korea 21 PLUS Project for Medical Science, Yonsei University College of Medicine, Seoul, 06229 Korea; 7 Department of Systems Biology, College of Life Science and Biotechnology, Yonsei University, Seoul, 06229 Korea; 8 KHU-KIST Department of Converging Science and Technology, Kyung Hee University, Seoul, 02447 Korea; 9 Department of Surgery, Yonsei University of College of Medicine, Seoul, 06229 Korea; 10 Present Address: RetiMark Co. Ltd., Seoul, 02792 Korea

**Keywords:** Molecular medicine, Colorectal cancer, Integrin signalling

## Abstract

The role of matrix metalloproteinase-2 (MMP-2) in tumor cell migration has been widely studied, however, the characteristics and effects of MMP-2 in clinical sample of metastatic colorectal cancer (CRC) remain poorly understood. Here, in order to unveil the perturbed proteomic signal during MMP-2 induced cancer progression, we analyzed plasma proteome of CRC patients according to disease progression, HCT116 cancer secretome upon MMP-2 knockdown, and publicly available CRC tissue proteome data. Collectively, the integrative analysis of multi-layered proteomes revealed that a protein cluster containing EMT (Epithelial-to-Mesenchymal Transition)-associated proteins such as CD9-integrin as well as MMP-2. The proteins of the cluster were regulated by MMP-2 perturbation and exhibited significantly increased expressions in tissue and plasma as disease progressed from TNM (Tumor, Node, and Metastasis) stage I to II. Furthermore, we also identified a plausible association between MMP-2 up-regulation and activation of focal adhesion kinase signaling in the proteogenomic analysis of CRC patient tissues. Based on these comparative and integrative analyses, we suggest that the high invasiveness in the metastatic CRC resulted from increased secretion of MMP-2 and CD9-integrin complex mediated by FAK signaling activation.

## Introduction

Colorectal cancer (CRC) is one of the most commonly diagnosed cancers and the third leading cause of cancer-related deaths worldwide. There are currently few treatment options for advanced and metastatic patients^1^. Although most primary CRC tumors can be resected surgically, CRC frequently spreads to other organs through metastasis and leads to high mortality^2^. Cancer metastasis is defined as a detachment of cancer cells from the primary tumor and subsequent infiltration to another part of the body. It is an irreversible and largely incurable stage of cancer progression and one of the leading causes of cancer death^3^. During the initial stage of metastasis, remodeling and path-clearing processes of the surrounding extracellular matrix (ECM) are activated^4,5^. Matrix metalloproteinase-2 (MMP-2), a zinc dependent endopeptidase, is one of the most important enzymes that participate in ECM organization and cell migration. A number of studies have found that elevated levels of MMP-2 determines the invasive and metastatic capacity of tumor cells^6–8^. In CRC, increased MMP-2 is also a key feature of highly metastatic tumors^4,5,9–11^. During invasion and metastasis, tumor cells not only secrete ECM degradative enzymes such as MMP-2, but also produce and secrete growth factors, cytokines, and hormones to entice host or neighboring cells into producing proteolytic enzymes to degrade the ECM^12–14^.

The secretome encompasses the total set of proteins that are actively transported to the outside of cells. It reflects the various pathological or physiological conditions of the cell^15^ and reflects pathological conditions such as cancer stage and metastasis in plasma^16^. As elevated level of MMP-2 promotes tumor progression and metastatic ability, the secretory proteins regulated by MMP-2 also could reflect the pathological status of cancer cell and be secreted to plasma proteome of CRC patients. However, the secreted proteins controlled by the genetic perturbation occurring during tumorigenesis or tumor migration are difficult to study in clinical samples from CRC patients due to genetic and physiological heterogeneity. Alternatively, the impact of altered expression of MMP-2 on the secreted proteins can be studied in a model system such as cancer cell lines. However, since almost all available CRC cell lines are obtained from malignant tumors, they do not represent proper models to investigate tumorigenesis according to phased cancer progression. Moreover, conditioned media harvested from cultured cells in vitro does not completely mimic that of patients’ blood samples, which contain secretory proteins from not only tumor cells, but also normal cells such as stromal cells or liver tissue. Thus, a secretome prepared in vitro can only provide an extremely simplified model compared with what actually occurs in vivo^17^. In spite of the significant advances in mass spectrometry, which enables an in-depth proteomic analysis of the secretome^15^, such an approach based only on cell lines may fail to validate results obtained from clinical samples.

To narrow this gap between studies of cultured conditioned medium and clinical material, we sought to integrate quantitative proteomics data from multiple sources: in vitro cultured cell line secretome and proteomes obtained from CRC patients; our own experimental data and proteomic data obtainable from public databases. We constructed an *MMP2* knockdown (KD) cell line, identified differentially secreted proteins, and integrated this secretome data into clinical proteomics data. By comparing quantitative information perturbed by *MMP2* KD and the proteomic expressional change according to tumor, node, and metastasis (TNM) stage, we suggest that increased metastatic feature of CRC during progression from stage I to II is the result of focal adhesion kinase (FAK) activation. Our understanding is also validated in this study in individual CRC proteogenomics data by various bioinformatic analysis.

## Results

### Plasma proteome profiling for differentiating the tumor stage of CRC

**Figure 1:**
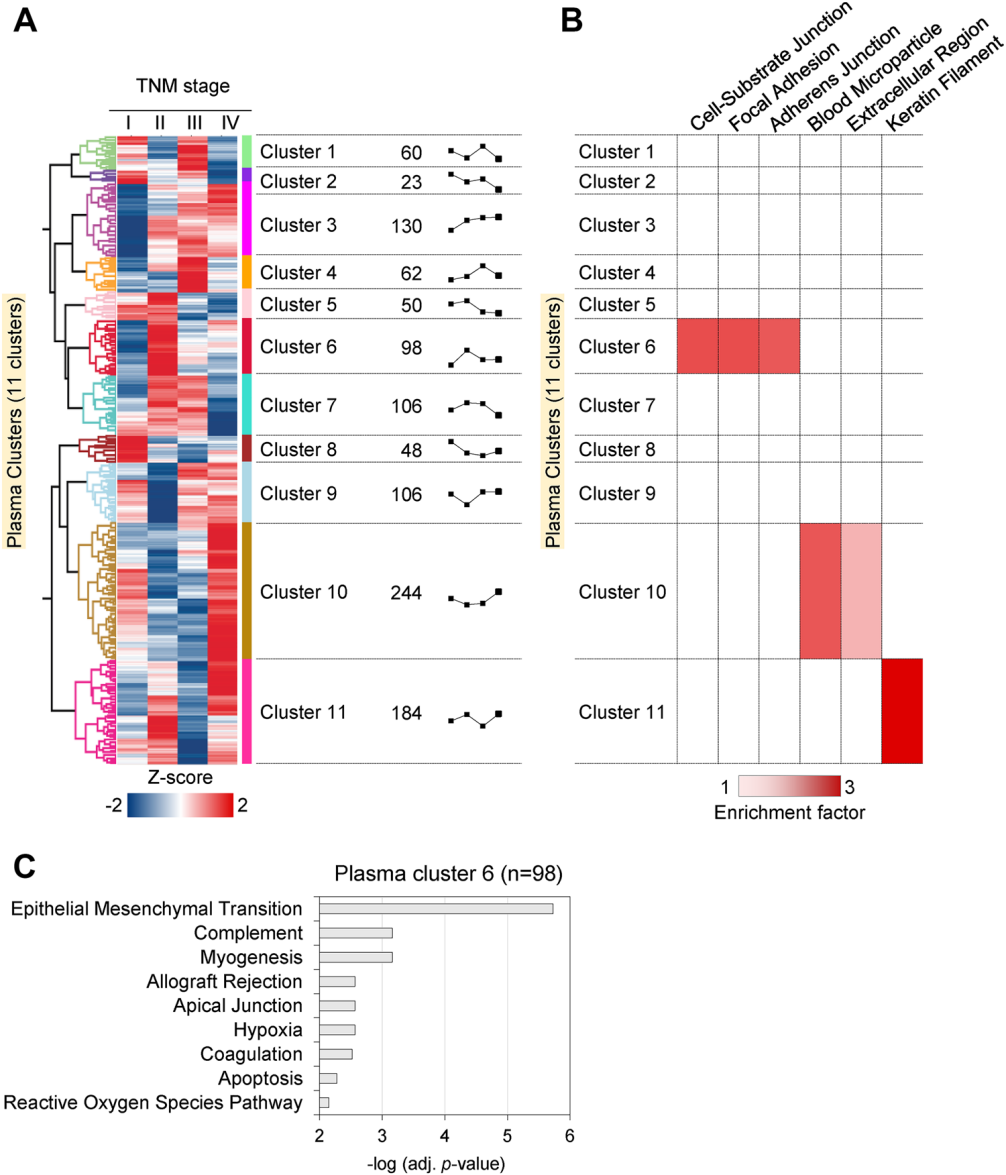
Plasma proteome analysis of CRC according to cancer stage. (**A**) Unsupervised hierarchical clustering analysis of CRC plasma proteins according to CRC stage was performed with Perseus software. Each of the 4 points in the graphic trend represents the averaged z-score of the protein expression level at the same CRC stage. (**B**) Fisher’s exact test between the 11 plasma clusters and GOCC value (Benjamini–Hochberg FDR < 0.02). Color intensity represents the enrichment factor for the association between the plasma cluster and GOCC value. (**C**) Functional enrichment of the plasma cluster 6 to hallmark gene sets from MSigDB (adjusted *p*-value < 0.01).

We first profiled the plasma proteins of CRC patients at different cancer stages as described in Fig. S1*A*. The classification of TNM stages of CRC patients was confirmed histologically and plasma samples representing the same TNM stage (I to IV) were each combined separately (Fig. S1*B*). The four different samples were then analyzed by label-free quantification (LFQ) proteomics after top12 abundant plasma proteins were depleted. A total of 1111 proteins were identified at 1% false discovery rate (FDR), 70% of which were identified in all samples with an average of 936 per sample (Table S1). Using the LFQ intensity, we classified plasma proteins based on their quantitative trend according to TNM stage. Unsupervised hierarchical clustering of the 1111 plasma proteins, after z-score normalization of the log2 LFQ intensities, resulted in 11 clusters (Fig. 1A). Figure 1A shows the average expression profile of each plasma proteome cluster (hereafter referred to as “plasma cluster”) according to TNM stage and the number of proteins belonging to the cluster. The plasma clusters were applied to the Fisher’s exact test to calculate functional enrichment of proteins belonging to each cluster as compared to all the proteins identified (Fig. 1B). Plasma cluster 10, which has the largest number of proteins, is related to the blood microparticle and extracellular region. Plasma cluster 11, which has the second-largest number of proteins, was enriched with keratin filament. Plasma cluster 6 containing proteins with radical protein increase from stage I to II was enriched with proteins of cell-substrate junction, focal adhesion, and adherens junction. As all the three terms enriched in plasma cluster 6 are associated to cell-to-cell adhesion complexes, we focused on this cluster and performed further bioinformatic analysis. Gene Set Enrichment Analysis (GSEA) on plasma cluster 6 using the hallmark gene sets from the Molecular Signatures Database (MSigDB)^18^ revealed the gene set “Epithelial and mesenchymal transition” significantly (*p*-value < 10^–5^, Fig. 1C). Epithelial and mesenchymal transition is a highly conserved cellular program that promotes metastasis. In the protein interaction network generated with plasma cluster 6, interactions were observed between 41 protein nodes, and MMP-2 was one of the key proteins connecting the largest number of proteins (Fig. S2). In our previous study, the expression level of MMP-2 was 3.03-fold higher in microsatellite stable type CRC tissues compared to matched normal tissues^19^. Consistent with our current result, it has also been reported that the highest MMP-2 activity is usually measured in stage II of CRC^20,21^. Therefore, we hypothesized that MMP-2 enhances the migration ability of CRC and a key modulator of tumor progression from cancer stage I to II.

### *MMP2* knock down impairs the migration ability of HCT116 CRC cells

**Figure 2:**
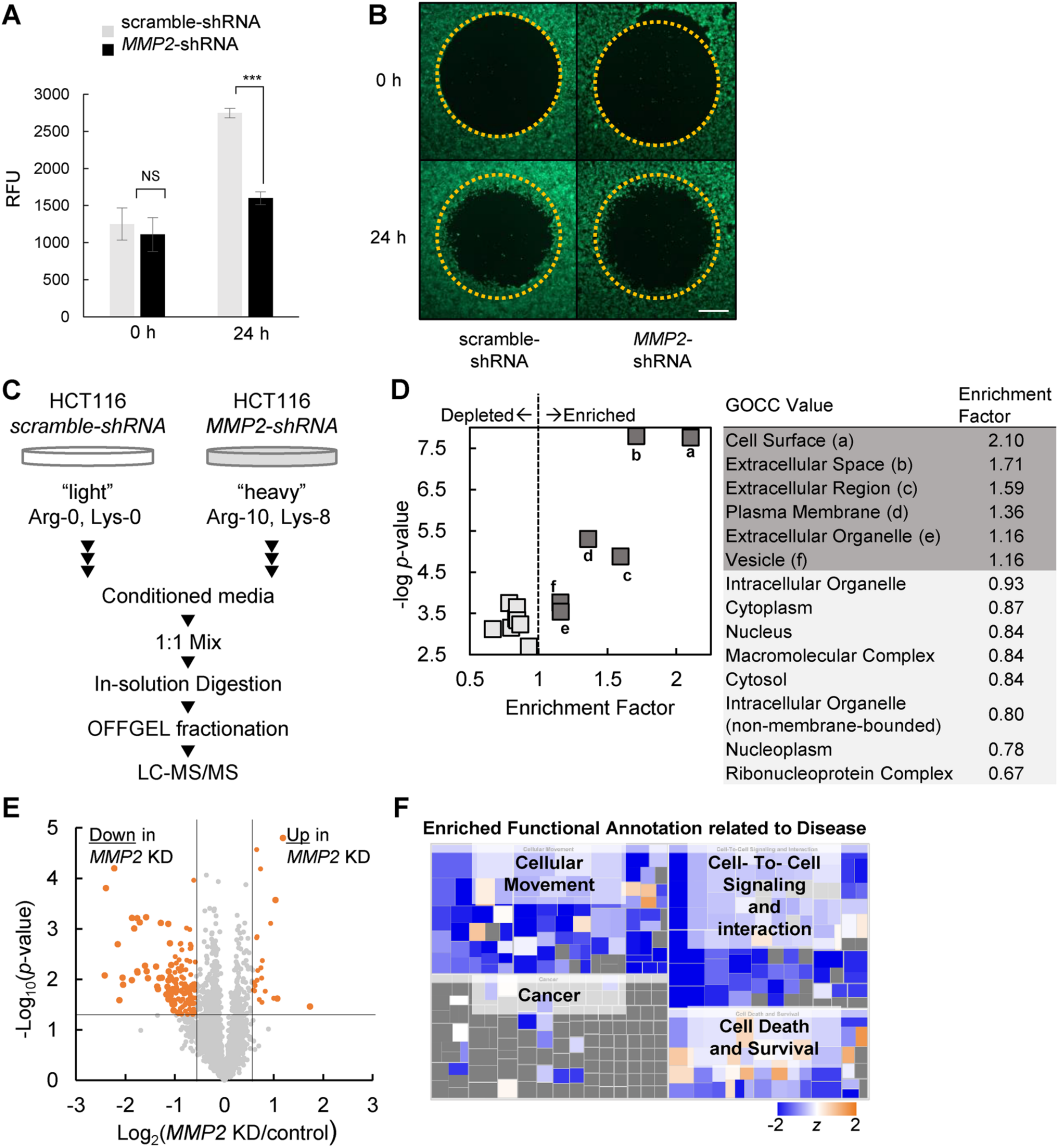
Analysis of differentially secreted proteins in HCT116 cells upon *MMP2* KD. (**A**) Quantification of cell migration of HCT116 cells upon *MMP2* KD. The error bars represent ± standard deviation (SD) (NS: not significant. ***: p < 0.001). (**B**) Representative fluorescence images of HCT116 cells showing cell migration activity at the indicated time after stopper. Dotted circles represent the initial stopper position. Scale bar, 500 µm. (**C**) Schematic workflow of the SILAC-based quantitative proteomic analysis of the HCT116 secretome. The experiments were performed three times with this setup and three more times with the reverse setup, i.e. HCT116 *MMP2* KD with light SILAC. (**D**) Gene ontology enrichment analysis was performed for the proteins identified as quantifiable at least three times out of six replicated SILAC experiments compared to all human proteins as the background list using Fisher’s exact test (Benjamini–Hochberg FDR < 0.02). (**E**) Volcano plot for the SILAC-quantitation values of the cell secretome. Orange dots indicate proteins with a Benjamini–Hochberg FDR < 0.05 and a fold change above ± 1.5. (**F**) Functional annotation of the differentially secreted proteins by using IPA. The blue color represents inactivation of the indicated annotation upon *MMP2* KD while the orange color represents activation. All quantified proteins were used as the background and Benjamini–Hochberg FDR < 0.02 was applied. Box size represents –log (*p*-value).

To evaluate the functional role of MMP-2 in CRC, we next investigated the effect of MMP-2 on migration ability of tumor in vitro cancer cell line. To this end, we initially designed a cell line that could serve as a model for highly metastatic CRC. HCT116, a human colon cancer cell line, has been shown to be invasive and highly motile in in vitro studies^22^. A stable cell line with attenuated MMP-2 activity was established by using an *MMP2* specific shRNA plasmid. A control cell line was also generated using a vector containing scrambled shRNA. There was no significant change in cell proliferation rate between the two cell lines (Fig. S3A). Quantitative PCR analysis confirmed that *MMP2* mRNA levels were 3.5-fold lower in the *MMP2* KD cells compared with the control cells (Fig. S3B). Decreased MMP-2 protein expression and proteolytic activity were also confirmed by western blot analysis and gelatin zymography, respectively (Fig. S3C-D). Cell migration ability, as measured by the area of migration, was significantly decreased in *MMP2* KD cells when compared with control cells (Fig. 2A,B). MMP-2 modulation of the tumor microenvironment has been studied extensively^23^. Together with our data, we hypothesized that MMP-2 also enhances the migration ability of CRC tumor cells by modulating the composition of the secretome.

### Differentially secreted proteins altered by MMP-2 are highly related to cell motility

In order to comprehensively characterize the proteins secreted into conditioned media, we performed a SILAC-based quantitative analysis on the secretome of both control (scramble-shRNA) and *MMP2* KD (*MMP2*-shRNA) cell lines as shown in Fig. 2C. We prepared three biological replicates from cells independently cultured with forward SILAC (Arg-0/Lys-0: light media for control and Arg-10 [^13^C_6_, ^15^N_4_]/Lys-8 [^13^C_6_, ^15^N_2_]: heavy media for *MMP2* KD) and three additional biological replicates cultured with reverse SILAC (light media for *MMP2* KD and heavy media for control) (Fig. S4*A*). Before sample preparation, near complete incorporation of SILAC amino acids into cellular proteins was confirmed (Fig. S4*B*). Conditioned media were harvested after serum free starvation for 24 h. Under these conditions, the cytoskeleton protein α-tubulin was scarcely detectable, implying negligible contamination of cytosolic proteins caused by cell death (Fig. S4*C*). As a result, we identified a total of 3196 human proteins including 99 FBS-derived bovine proteins and 13 ambiguous proteins, in which the origin could not be explicitly. Among the 3196 human proteins, 2596 (81%) were quantifiable; 1972 in at least three sets from SILAC analysis and 1278 in all six sets (Table S2).

The quality of our secretome was assessed by Gene Ontology Cellular Compartment (GOCC) enrichment analysis for the proteins quantifiable in all six secretome datasets. Significantly enriched GOCC terms, having an enrichment factor greater than one in Fisher’s exact test, were all strongly associated with extracellular localization. GOCC depleted terms having an enrichment factor < 1 were associated with cytoplasmic localization (Fig. 2D). Collectively, the GO analysis strongly indicated that we could exclude the most frequent, technically-biased identifications that occur due to contamination by FBS proteins and cellular debris.

The quantitation results exhibited a typical normal distribution when log-transformed protein ratios were plotted as histograms (Fig. S5) and in a volcano plot (Fig. 2E). By setting a 1.5-fold change as the threshold and a *p*-value < 0.05 for significant up- or down- regulation of the proteins, we identified 164 differentially expressed proteins (DEPs). Of those, 142 proteins exhibited down-regulated expression and 22 proteins exhibited up-regulated expression (Fig. 2E). Gene ontology for molecular function (MF) and biological process (BP) for the DEPs was analyzed using Perseus (Table S3). The GOBP gene sets enriched in a Fisher’s exact test were involved in “extracellular matrix (or structure) organization,” “cell–cell signaling” and “cell motility.” Likewise, enriched GOMF terms were mostly related to peptidase activities. Ingenuity pathway analysis (IPA) was also performed on the DEPs to enrich disease-related functional annotations and to predict the direction of activation. The analysis indicated “cellular movement,” “cell to cell signaling” and “cell death” as the most enriched annotations and these activities were predicted to be suppressed by *MMP2* KD (Fig. 2F).

### Comparison of cell secretome to plasma proteome profile of cancer patients confirms a consistent regulatory effect of MMP-2 with disease progression

**Figure 3:**
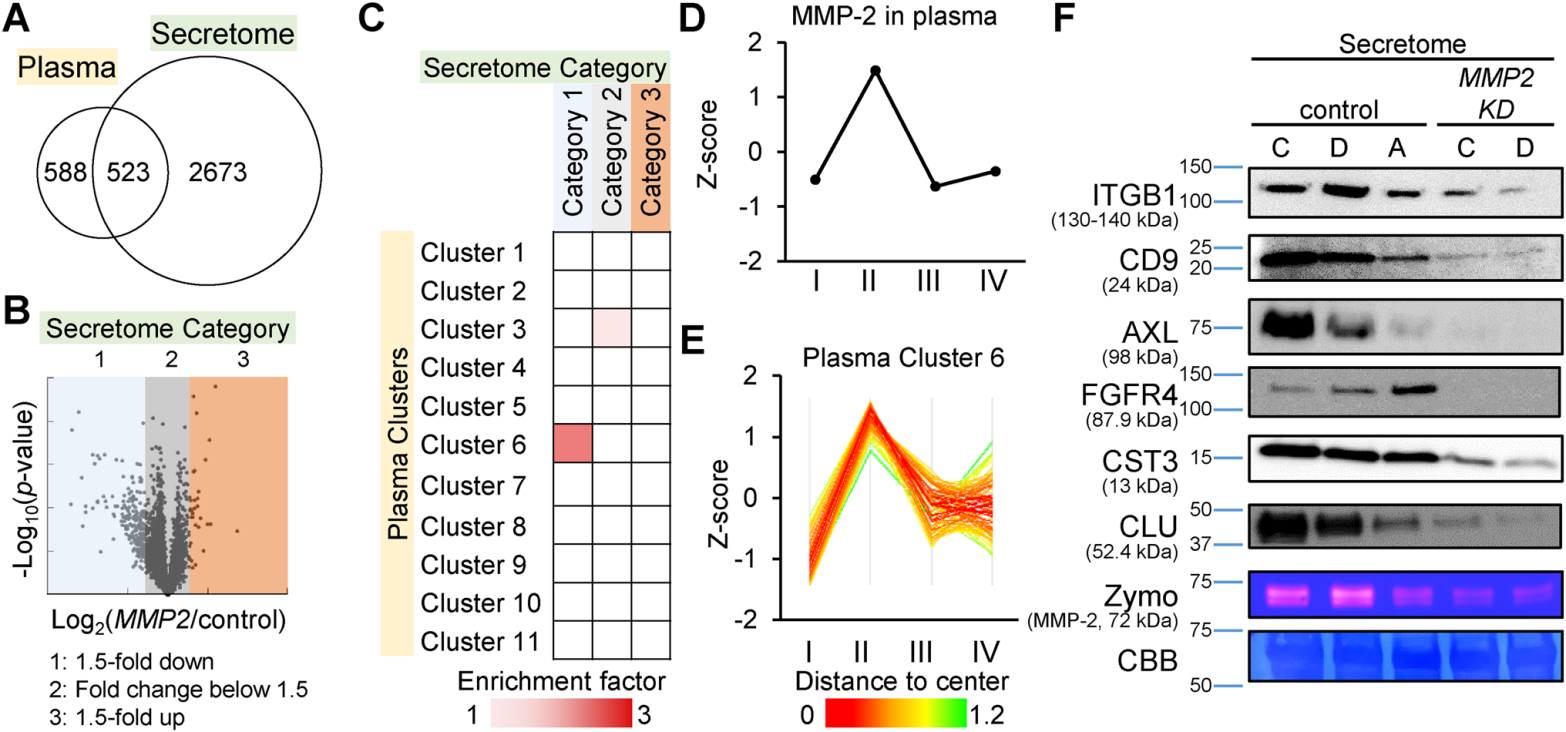
Comparative analysis of the cell secretome with the plasma proteome of CRC cancer patients. (**A**) The numbers in the Venn diagram represent those of identified proteins. (**B**) The HCT116 secretome was divided into 3 categories based on log_2_(*MMP2* KD/Control) ratio. (**C**) Fisher’s exact test between the three secretome categories and 11 plasma clusters (Benjamini–Hochberg FDR < 0.02). Color intensity represents the enrichment factor for the association between the secretome and plasma clusters. (**D**) Z-scored expression profile of MMP-2 in plasma proteome according to CRC stage. (**E**) Z-scored expression profiles of proteins in the plasma cluster 6. (**F**) Western blot analysis of the proteins commonly identified in both secretome category 1 and plasma cluster 6. Protein samples were prepared from the conditioned media (10 µg) of HCT116 Control and HCT116 *MMP2* KD. *C* Control, *D* DMSO-only, *A* ARP-100 treated. Full-length blots are available in supplemental information.

In order to determine the disease-association of the secretory proteins that were strongly regulated by MMP-2, we next performed a comparative analysis on the two proteomes, secretome and plasma proteome. The HCT116 secretome data contained 523 matches commonly identified with the plasma proteome (Fig. 3A). As we classified the plasma proteome into multiple clusters, we also grouped the secretome into three categories based on the fold change ratio: category 1 for proteins decreased > 1.5-fold in *MMP2* KD; category 2 for proteins with less than 1.5-fold change and category 3 for proteins increased > 1.5-fold in *MMP2* KD secretion (Fig. 3B). The common proteins between the plasma proteome and secretome were evenly populated among the 11 clusters and present in similar proportions in each cluster (Table S4). However, if we further classified the proteins into subgroups according to secretome categories, non-random associations between the two different proteomes appeared. Fisher’s exact test was performed to determine if there were non-random associations between the plasma clusters and secretome categories (Fig. 3C). Plasma cluster 6 exhibited a strong association with secretome category 1 with an enrichment factor of 2.27 (*p* = 6.5 × 10^−4^). Another significant association was found between plasma cluster 3 and secretome category 2 (enrichment factor = 1.23, *p* = 5.9 × 10^−3^). Intriguingly, the plasma cluster 6 contains MMP-2, which we specified as the knockdown target through a functional enrichment analysis (Fig. 1B). This suggests that there is an increase in the secretion of proteins commonly present in plasma cluster 6 and secretome category 1 from colon epithelial cells when MMP-2 expression increases (Fig. 3D,E). There were a total of 16 common proteins, which may be regulated by or at least associated with MMP-2 (Table 1).

**Table 1:** List of the 16 proteins that show decreased secretion by MMP2 KD in CRC cells and increased levels in the plasmas of CRC patients during disease progression from stage I to II.

Accession No	Protein name	Gene name	Plasma (LFQ)				Secretome (SILAC)	
			Z-score				Log_2_ (*MMP2-KD*/ control)	*p*-value
			CRC I	CRC II	CRC III	CRC IV		
P05556	Integrin beta-1	ITGB1	− 0.77	1.46	− 0.54	− 0.15	− 0.97	0.005
P21926	CD9 antigen	CD9	− 0.81	1.26	− 0.82	0.37	− 0.70	0.016
Q86SQ4	G-protein coupled receptor 126	GPR126	− 0.72	1.49	− 0.36	− 0.41	− 1.02	0.03
P20827	Ephrin-A1	EFNA1	− 1.06	1.27	− 0.47	0.27	− 1.00	0.016
Q15828	Cystatin-M	CST6	− 1.02	1.36	− 0.37	0.03	− 0.97	0.045
P30530	Tyrosine-protein kinase receptor UFO	AXL	− 1.38	1.02	0.3	0.07	− 0.89	0.039
P22455	Fibroblast growth factor receptor 4	FGFR4	− 0.61	1.46	− 0.7	− 0.17	− 0.80	0.033
P43121	Cell surface glycoprotein MUC18	MCAM	− 0.6	1.48	− 0.66	− 0.23	− 0.76	0.005
Q14118	Dystroglycan	DAG1	− 0.45	1.5	− 0.4	− 0.66	− 0.66	0.043
P98160	Basement membrane-specific heparan sulfate proteoglycan core protein	HSPG2	− 1.03	1.3	0.22	− 0.49	− 0.64	0.049
P10316	HLA class I histocompatibility antigen	HLA-A	− 1.21	1.16	− 0.32	0.37	− 0.74	0.052
P01034	Cystatin-C	CST3	− 1.37	0.92	− 0.1	0.55	− 0.72	0.06
P39060	Collagen alpha-1(XVIII) chain;Endostatin	COL18A1	− 0.94	1.41	− 0.4	− 0.08	− 0.74	0.064
O00592	Podocalyxin	PODXL	− 1.33	1.1	− 0.03	0.26	− 0.62	0.09
P10909	Clusterin	CLU	− 1.4	0.99	0.24	0.19	− 0.85	0.092
Q99715	Collagen alpha-1(XII) chain	COL12A1	− 1.25	1.18	0.21	− 0.14	− 0.60	0.122

We then performed immunoblotting as an orthogonal validation method (Fig. 3F) to confirm the regulative expression of the 16 proteins identified from the SILAC results. We confirmed that most of the 16 proteins exhibited decreased expression in the secretome of the *MMP2* KD cell line, and also in the normal secretome treated with ARP-100, an MMP-2 specific irreversible inhibitor. Overall, our results suggest that the integration of multidimensional data from cell lines perturbed at a transcription level, and data from clinical samples reflecting similar pathological conditions, can offer better prospects to find a novel signature of cancer etiology.

### Comparison of the secretome, plasma proteome, and tissue proteome reveals the CD9-integrin complex as key molecules for CRC

**Figure 4:**
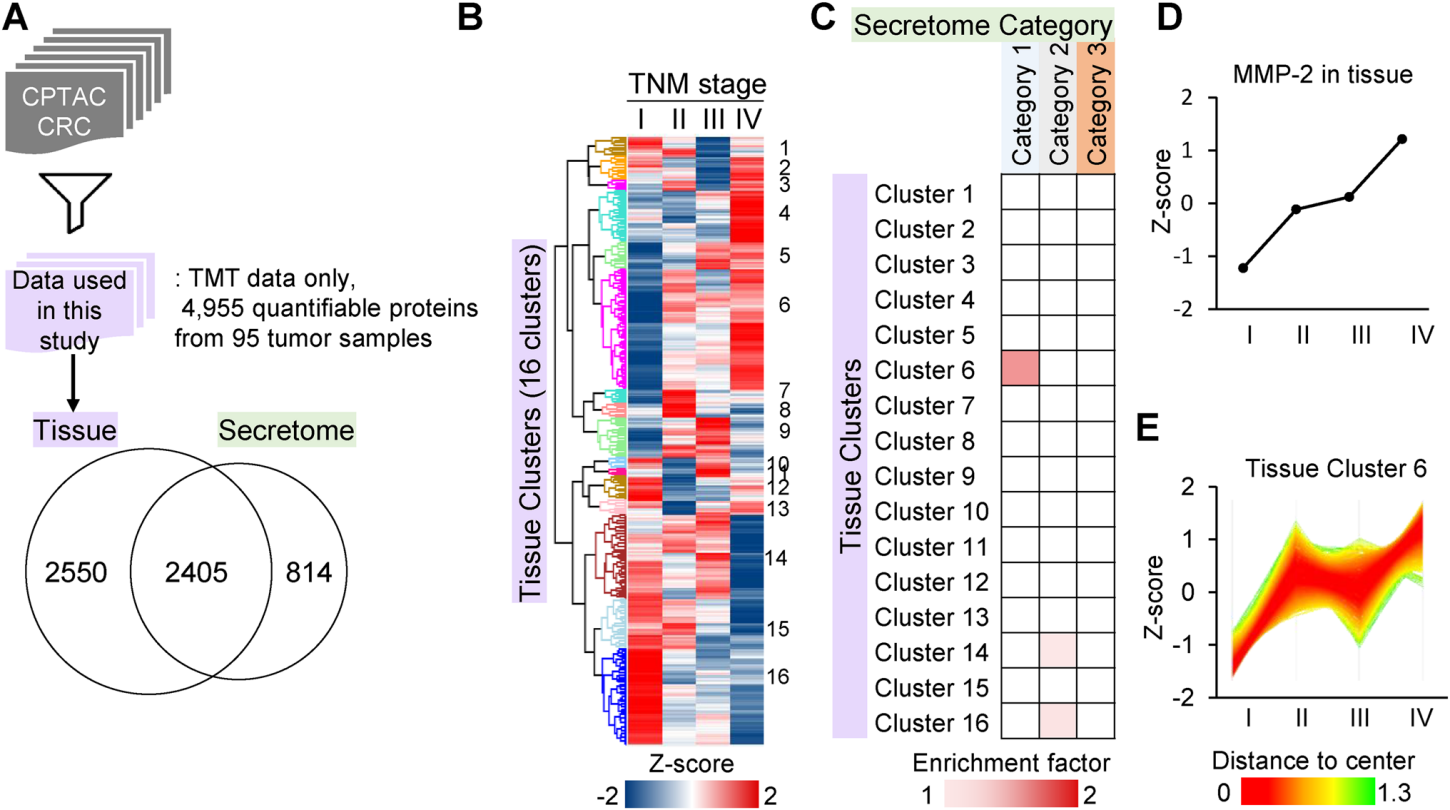
Comparative analysis of the cell secretome with the tissue proteome of CRC cancer patients. (**A**) Schematic workflow of tissue proteome analysis. The tissue proteomic data were obtained from a public data repository shared by CPTAC. The numbers in the Venn diagram represent overlap between identified proteins from the HCT116 secretome and the tissue proteome. (**B**) Unsupervised hierarchical cluster analysis of CRC tissue proteins according to CRC stage. (**C**) Fisher’s exact test between the three secretome categories and 16 tissue clusters (Benjamini–Hochberg FDR < 0.02). The color intensity represents the enrichment factor of the association between the secretome and tissue clusters. (**D**) Z-scored expression profile of MMP-2 in the tissue proteome relative to CRC stage. (**E**) Z-scored expression profiles of the proteins in the tissue cluster 6.

The next step compared concordance between the plasma proteome profile with the tissue proteome profile obtained from CRC patients. For this, we downloaded publicly available data from Clinical Proteomic Tumor Analysis Consortium (CPTAC, NCI/NIH; see Table S5 for detailed clinical information) and collected quantitation data for 4,955 proteins. Of these, 2,409 proteins were common to that identified in our SILAC secretome data (Fig. 4A). We applied the same clustering strategies to this tissue proteome data and performed a comparative analysis between the patient-derived tissue proteome and cell line-derived secretome. Unsupervised hierarchical clustering of 4,955 tissue proteomic elements using relative expression profiles associated with TNM stage revealed 16 tissue proteomic clusters (hereafter referred to as “tissue cluster”, Fig. 4B, Table S6). A cluster named tissue cluster 6 showed a strong association with category 1 of the secretome as determined by Fisher’s exact test (*p*-value = 2.7 × 10^−3^, enrichment factor = 1.55; Fig. 4C). The cluster shared 408 proteins in common with the secretome data. Of these 408 proteins, 31 proteins showed decreased expression of more than 1.5-fold when *MMP2* was knocked down. Interestingly, we also observed MMP-2 in this subgroup. This is consistent with the comparative analysis result between the secretome and the plasma proteome as described above. Expression of MMP-2 in the tumor samples increased concomitantly with the progression of CRC clinical stage (Fig. 4D). Differences in MMP-2 expression between CRC stages I and II in individual tissue proteomes were statistically significant (Figure S6). When we compared the expression profile of the plasma cluster 6 (Fig. 3E) and the tissue cluster 6 (Fig. 4E), both showed the most drastic increase in the progression from CRC stage I to II.

**Figure 5:**
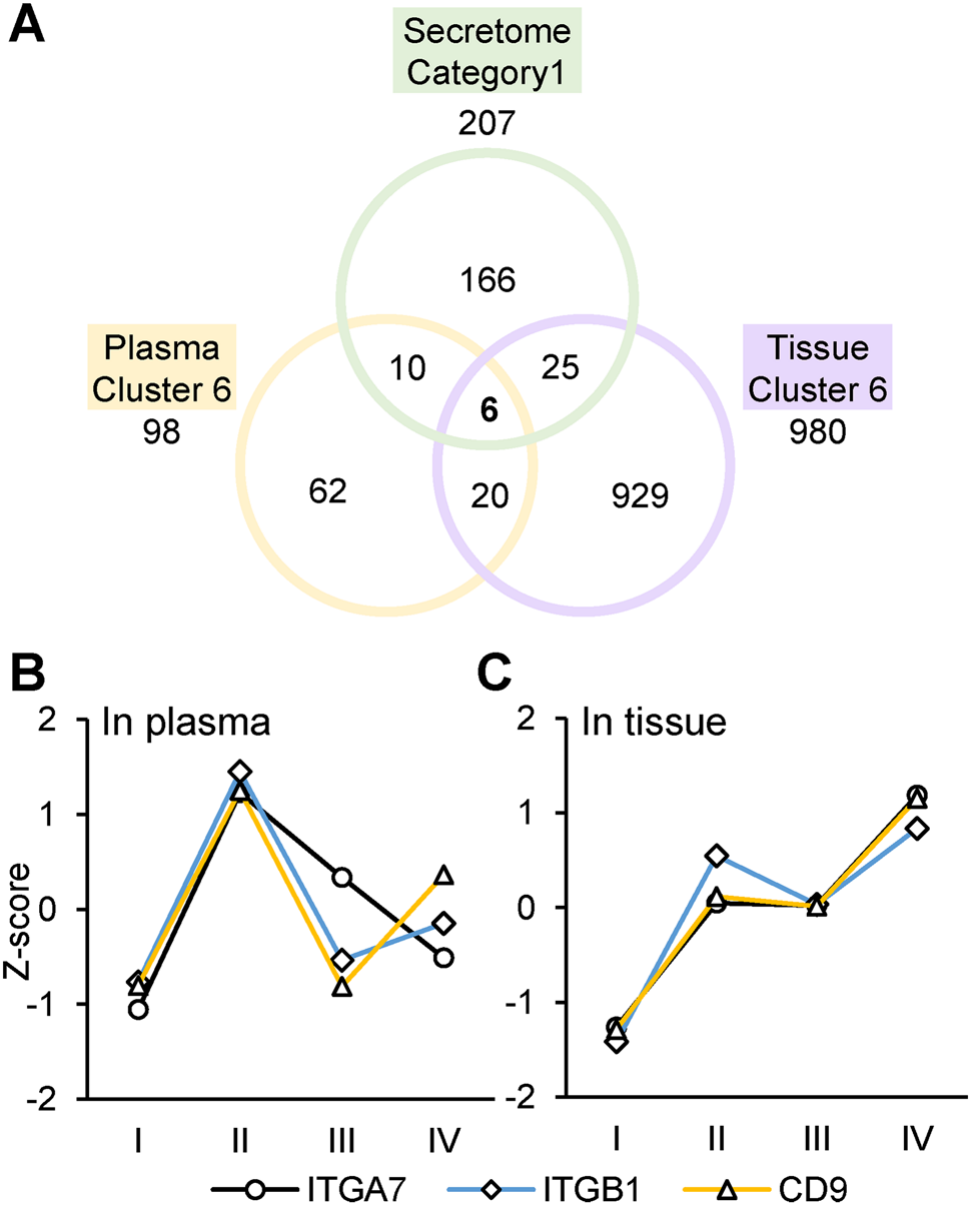
Overview of the proteins highly correlated with MMP-2 perturbation and disease progression. (**A**) The multi-layered proteome analysis result is presented as a Venn diagram depicting overlap between the secretome category 1, plasma cluster 6, and the tissue cluster 6. (**B**,**C**) Expression profile of the ITGA7-ITGB1-CD9 complex in plasma (**B**) and tissue (**C**) of CRC patients relative to cancer progression.

We found 16 common proteins that were enriched from the comparison of the SILAC secretome to the CRC plasma proteome. We also found 31 common proteins that were enriched from the comparison to the tissue proteome. In these two comparative analyses, 6 proteins were found in common: CD9, ITGB1, HSPG2, CLU, COL18A1, and COL12A1 (Fig. 5A). The surface proteins CD9 and ITGB1 are known to form a complex with ITGA7^24^ and play a key role in the modulation of integrin-dependent cell migration and adhesion strengthening^25^. In our data sets, all three components of this complex were found in the same clusters within the plasma and tissue proteomes. This finding implies that the components of ITGA7-ITGB1-CD9 complex represent similar expression profiles and associated with tumor progression (Fig. 5B,C). Although ITGA7 was not identified in our secretome analysis, the expression, or secretory regulation of the complex is likely to be related to MMP-2.

### MMP-2 regulates cell migration via the FAK pathway

**Figure 6:**
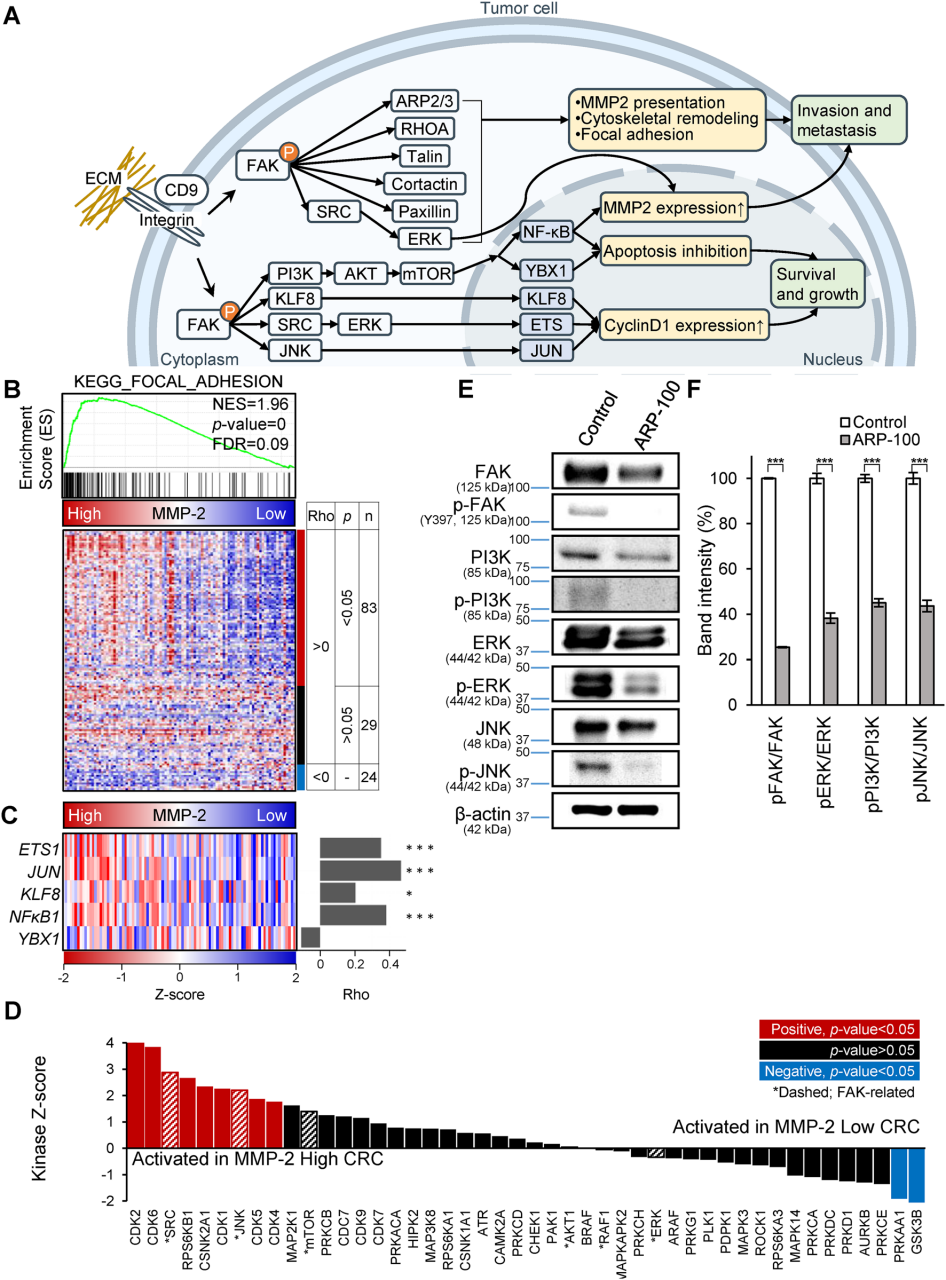
Regulation of FAK signaling by MMP-2 activity. (**A**) The focal adhesion kinase pathway. FAK activated by integrin-CD9 complex increases cell motility and cell survival. (**B**) GSEA of CPTAC CRC patients showed that focal adhesion pathway is associated with the MMP-2 high group (MMP-2 protein expression is higher than median level). Heat map represents the relative protein abundance of 136 genes associated in focal adhesion pathway. Correlation test was performed between the expressions of MMP-2 and each of 136 genes. 83 proteins show significant and positive correlations to MMP-2 expression (Spearman’s correlation coefficients (rho) > 0, *p*-value < 0.05). (**C**) Correlation between MMP-2 and transcriptional factors in FAK signaling pathway in the CPTAC CRC data. Heatmaps for MMP-2 represents protein abundance. Heatmaps for ETS1, JUN, KLF8, NFKB1 and YBX1 represent transcription factor activity scores. Bar charts on the right represent Spearman correlation coefficients (rho). *P < 0.05, **P < 0.01; Spearman correlation test. (**D**) Evaluation of kinase activity by KSEA in MMP-2 high group and MMP-2 low group. Asterisks indicate the kinases associated in FAK signaling pathway. (**E**) Phosphorylation of FAK, ERK, PI3K, JNK were measured by western blot analysis with corresponding phospho-site-specific antibodies. Protein samples were prepared from the cell lysate of each condition and 25 µg of proteins were separated on SDS-PAGE. Full-length blots are available in supplemental information. (**F**) The phosphorylation level was determined by normalized phospho-band intensity of each protein against the total protein band intensity in (**E**). The error bars represent ± SD of three independent experiments.

The selected findings were further investigated for biological significance. It is well known that activation of FAK signaling involves integrin receptor clustering upon binding of cells to ECM proteins^26^. The FAK signaling pathway represents a major regulatory pathway that receives numerous extracellular stimuli, which are and transmitted intracellularly to control cell adhesion and motility (Fig. 6A)^26,27^. Since we found correlative expression profiles between MMP-2 and the CD9-integrin complex in all three kinds of proteome data, we further examined whether the activation of FAK signaling was also related to MMP-2 expression level. Furthermore, we also analyzed other proteogenomic data (transcriptome and phosphoproteome) of the same CPTAC CRC cohort from which the tissue proteome was obtained.

First, we divided the 95 CPTAC CRC patients into two groups based on their expression level of MMP-2 (Top 50%: MMP-2 high, n = 47; bottom 50%: MMP-2 low, n = 48). GSEA using quantifiable 4,955 tissue proteins revealed that KEGG focal adhesion pathway was significantly enriched in the MMP-2 high group (Fig. 6B). Among 136 proteins included in the focal adhesion pathway, 83 proteins (61%) showed strong positive correlation with MMP-2 expression level across the patients (Spearman’s rank correlation coefficient > 0, *p*-value < 0.05).

We also evaluated that FAK signaling event at mRNA expression level. It has been well known that there are several transcription factors (TFs) are induced by FAK^26^. FAK induces cell cycle progression and apoptosis inhibition through transcriptional regulation by KLF8, ETS, JUN, NFκB and YBX1 (Fig. 6A). As a way of showing induction of transcription factors, we calculated activation score of TFs. The activation score of TFs was calculated based on the transcriptional abundance of their known target genes^28^. As a result, the activation scores of TFs except YBX1 were positively correlated with the protein expression level of MMP-2 (Fig. 6C, Spearman’s rank correlation coefficient > 0, *p*-value < 0.05).

When integrin receptor clustering bind to extracellular matrix (ECM), it activates FAK and the activated FAK induces downstream pathway mediated through phosphorylation cascade^26,27^. Therefore, we would like to find an evidence at the phosphoproteome level related to FAK activation. Kinase substrate enrichment analysis (KSEA) on the phosphoproteome of MMP-2 high group against MMP-2 low group identified multiple kinases such as SRC, JNK and mTOR, which are known to be associated in FAK signaling pathway and to be activated in tumors (Fig. 6D).

To further investigate the regulation of FAK signaling upon MMP-2 expression in HCT-116 CRC cell line, we measured the phosphorylation status of FAK, ERK, PI3K, and JNK by western blot analysis. Phosphorylation levels of these FAK signaling proteins were all decreased when cells were treated with 100 µM of ARP-100, an MMP-2 inhibitor (Fig. 6E,F). Protein levels also decreased, though not as their much as phosphorylation levels.

## Discussion

In this study, we applied a bioinformatics approach to assemble proteomic data from multiple CRC sources and establish consensus observations between proteome changes resulting from clinical differences to genetic perturbation. For this, we searched for proteins not only with differential secretion patterns according to altered expression of MMP-2 in HCT116 CRC cells, but also showing different levels in the plasma and tissue of CRC patients relative to cancer stage. The rationale of this strategy was that proteins associated with MMP-2 could be key mediators of CRC metastasis. In human cancer, tumor cells are not the dominant contributor of MMP-2 secretion to the ECM. Stromal cells and infiltrated immune cells also secrete MMP-2^29–31^. Irrespective of its origin, MMP-2 degrades the ECM and thus induces remodeling of the matrix and subsequent migration of the tumor cell^32^. Since MMP-2 is highly expressed in cancer cells, we opted to suppress MMP-2 instead of overexpressing it in HCT116 cells to create a less invasive cell. In fact, blocking MMP-2 translation led to decreased cell migration and perturbed the secretome, which was associated with cell migration and ECM organization as determined by gene ontology analysis. This finding is consistent with that of several previous studies in which MMP-2 amplified the motility of cancer cells^13,14^. Moreover, the molecular function of both increasing and decreasing proteins was primarily peptidase activity. This suggests that MMP-2 modulates the tumor microenvironment by itself and by regulating various proteases and peptidases^23,33–35^.

In order to connect the information obtained from different analyses, we classified proteins in each proteome dataset by expression pattern according to cancer stage, and then search for protein groups in which specific proteins were repeatedly found. Interestingly, the only one such protein group included MMP-2 and showed a similar expressional pattern to MMP-2. This suggests that a phenomenon found in clinical samples from CRC patients can be recapitulated by modulating MMP-2 expression and activity in a cell line. It is intuitive that an in vitro study, which may be readily controlled, does not accurately reflect the in vivo environment. Thus, experimental results from the laboratory do not always applicable to the clinic. However, we showed that a comparative study at the proteomic level between in vitro genetically perturbed cells and clinical samples may be effective at narrowing this gap. The proteins included in both the plasma and tissue clusters that had a high correlation with the perturbed secretome, showed a dramatic increase as the disease progressed from stage I to II. It has been reported that the highest MMP-2 activity is usually measured in stage II of CRC^20,21^. In the clinic, cancer progression is determined by a staging system, which reflects the extent, or metastasis at the time of diagnosis. The current system for CRC staging is the TNM system, which is based on the size or extent of the primary tumor and whether the tumor cell has migrated to nearby lymph nodes or other organs. Stage II CRC is particularly distinguishable from stage I according to whether the tumor has spread through the muscle layer of the colon wall to the serosa^1,36^. Our results suggest that robust mobility is required for tumor cells to penetrate the muscle layers. Thus MMP-2 plays a major role for such invasiveness in CRC during progression from stage I to II.

Among the proteins revealed by the comparative analysis, we focused on the CD9-ITGB1-ITGA7 complex. As we described in Fig. 6A this complex has been known to initiate FAK signaling by transmitting signals intracellularly^25,27^. The cytosolic protein FAK can interact directly with the integrin cytoplasmic tail, thereby allowing integrins to link to the actin cytoskeleton and promote downstream signaling^37^. CD9 also interacts with the phosphorylated form of FAK (Tyr397), a modification stimulated by integrin clustering^38^. CD9 is a member of the tetraspanin superfamily, which is characterized by an ability to associate with various transmembrane proteins forming tetraspanin-enriched microdomains^39^. Previous reports have shown that tetraspanin CD9 can either promote or suppress cancer cell migration and metastasis, depending on the type of interacting partner, cancer type, cell type, or the migratory signal^40–45^. Other studies have shown that CD9 expression affects MMP-2 secretion in various disease models^46–48^, and MMP-2 secretion increases as a result of FAK signaling activation^49^.We observed significant downregulation of CD9 and ITGB1 in the secretome of MMP-2 suppressed cells. These proteins showed a similar expressional trend as MMP-2 in both plasma and tissue proteomes according to CRC stage. Although the third component of the complex, ITGA7 was not identified in the cell secretome, it was found in the same plasma and tissue clusters where the other two components were found. Thus, it is likely that the CD9-ITGB1-ITGA7 complex as a whole play a role in cancer progression.

We have conducted extensive study of MMP-2 and FAK signaling pathway in the context of proteogenomics data from CPTAC CRC^50^. It is observed that, in CRC patient, FAK signaling would be more active as the expression level of MMP-2 increased. Based on transcriptome level, TFs known to be activated by FAK, showed higher transcriptional activity in MMP-2-high groups as measured based on transcriptome level of downstream targets. Moreover, at proteome level, FAK signaling pathway was more activated in the MMP-2 high-group than in the MMP-2 low group as abundance of most proteins associated in FAK signaling showed significantly positive correlation with the abundance of MMP-2 across the CRC patients. It is plausible that activation of FAK signaling also increased the MMP-2 expression as FAK also activates the SRC-ERK signaling, and ERK induces the expression of MMP-2^26,51,52^. FAK has kinase functions and transfers signals from extracellular space to nucleus via phosphorylation mediated signal cascade^53^, however, phosphopeptides of the direct substrate of FAK were detected only in a few samples of CPTAC cohort, making it difficult to determine the statistically significant level of phosphorylation. Nevertheless, based on phosphorylation status of indirect substrate of FAK signaling pathway, multiple kinase including SRC, mTOR and JNK exhibited the increased kinase activity score in MMP-2 high group.

Our western blot experiment further indicated that secretion of the complex depends on the protease activity of MMP-2, because treatment with a catalytic inhibitor exhibited a similar effect to genetic suppression. This demonstrates that MMP-2 activates FAK signaling in an activity-dependent manner. We observed an inhibition of MMP-2 activity and a reduction in FAK autophosphorylation (p-FAK-tyr397), an essential indicator of the integrin mediated FAK signaling activation^54^. We also observed that proteins such as ERK, PI3K, and JNK in the downstream FAK signaling cascade were also inactivated. FAK pathway is located at the central hub in various signaling pathways, controls cell motility by activating transcription factors of related genes^55–57^. It has been reported that the activated FAK causes epithelial-mesenchymal transition (EMT) in cancer metastasis in colorectal cancer cells and thereby enhancing the invasion and metastasis of colorectal cancer^58^. Therefore, these results support a hypothesis that increased MMP-2 activity enhances cellular motility by forming a positive feedback loop with the CD9-integrin complex and FAK signaling pathway.

In summary, we identified MMP-2 regulatory proteins by collecting concordant evidence between the secretome of HCT116 colon cancer cells, the CRC patient plasma, and tissue proteomes. The biological function of those proteins showed significant correlation in such comparative analysis and was highly associated with cell migratory ability. Their expression level also increased sharply when CRC progressed from stage I to II. We demonstrated that high invasiveness, featured in CRC cells with increased secretion of MMP-2, is the result of MMP-2 activation of FAK signaling through the CD9-integrin complex. Consequently, this induces a positive feedback of MMP-2 upregulation. The experimental approach performed here will provide a new strategy for the discovery of diagnostic and prognostic markers, which may increase the relative success of translational research.

## Materials and methods

### Plasma proteome analysis

#### Preparation of plasma samples

The plasma samples used in this study were collected from 187 CRC patients who had been recruited by the Yonsei Severance hospital (Seoul, Korea) for our previous study^59^. All participating patients were full informed and informed consent was obtained from each patient. Authorization to use these samples for research was obtained from the Institutional Review Board of the Yonsei Severance hospital. For all blood preparation, 3 mL of blood was collected in an EDTA tube, and the plasma was prepared as suggested by Human Proteome Organization Plasma Proteome Project^60^. The number of CRC patient donors in TNM stages I, II, III, and IV were 48, 48, 64, and 27, respectively. The plasma samples were combined according to TNM stage. The pooled plasma samples were then mixed with a protease inhibitor cocktail (complete Mini EDTA-free, Roche), aliquoted and immediately frozen at − 80 °C. All experiments performed in this study were carried out in accordance with the approved guidelines and regulations.

#### Depletion of high abundance proteins

An affinity-based depletion spin column (Thermo Scientific Pierce) was used for single-step removal of twelve high abundance proteins. These top twelve abundant proteins were albumin, transferrin, fibrinogen, α_1_-antitrypsin, haptoglobin, α_1_-acid glycoprotein, α_2_-macroglobulin, apolipoprotein A-I, apolipoprotein A-II, IgA, IgG, and IgM. Pooled plasma (10 µL) was loaded directly to the resin slurry in a spin column. A total of 40 µL of each plasma sample from each group was depleted. The mixture in the column was incubated with end-over-end mixing for 1 h at RT. After incubation, the unbound fraction was collected and concentrated by ultrafiltration using Amicon Ultra-0.5 Centrifugal filters (3 kDa cutoff; Millipore, MA), exchanged to a buffer containing 8 M urea, 75 mM NaCl and 50 mM Tris–HCl (pH 8.2)^61^. Protein concentrations were determined using a bicinchoninic acid assay kit (Thermo scientific, Rockford, IL).

#### Digestion of plasma proteins

The depleted plasma samples (150 µg) were reduced with 5 mM TCEP at 25 °C for 1 h and alkylated with 15 mM iodoacetamide at 25 °C for 1 h in the dark. The samples were diluted tenfold with 50 mM Tris–HCl (pH 8.2) and treated with mass spectrometry grade mixed trypsin/Lys-C (Promega, Madison, WI) at 37 °C for 16 h. The ratio of enzyme to protein was 1:25 (w/w). The digest was desalted using a C18 macrospin spin column (Harvard Apparatus, MA, USA), vacuum-dried and stored at − 20 °C until use.

#### Basic pH reversed-phase liquid chromatography

Peptides were fractionated by basic pH reversed-phase liquid chromatography using an Agilent 1290 Infinity LC System (Agilent Technologies). The chromatography was performed on an XBridge BEH130 C18 column (4.6 μm i.d. × 250 mm length; pore size 130 Å and particle size 3.5 μm; Waters Corporation, Milford, MA, USA) at a flow rate of 0.5 mL/min^62^. For mobile phase, 10 mM ammonium formate (pH 10) was set as channel A and 10 mM ammonium formate (pH 10) in 90% acetonitrile (pH 10) as channel B. The peptides were dissolved in 125 μL of mobile phase A and then injected into a 100 μL sample loop. Gradient setup was 2–5% B for 10 min, 5–40% B for 40 min, 40–70% B for 15 min, holding 70% B for 10 min, and finally 70–5% for 15 min. Fractionation was done by collecting 96 tubes (1 tube/0.8 min) throughout running. The resultant 96 fractions were pooled to 12 concatenated fractions by the following rule. A set of an arithmetic sequence with a common difference of 12 was pooled into one concatenated fraction, for instance, number 1, 13, 25, 37, 49, 51, 63 and 75 fractions were pooled into the first concatenated faction. The fractions were vacuum-dried and stored at –20 °C until MS analysis.

#### Liquid chromatography and tandem mass spectrometry (LC–MS/MS)

LC–MS/MS was performed, as previously described^63^. Peptides were reconstituted in 0.4% acetic acid and analyzed on a reversed-phase C18 column (20 cm × 75 μm i.d., 3 μm, 120 Å, packed in-house; Dr. Maisch) using an Eksigent nanoLC-ultra 1D plus system at a flow rate of 300 nL/min. Before use, the column was equilibrated with 95% buffer A (0.1% formic acid in H_2_O) and 5% buffer B (0.1% formic acid in acetonitrile). The peptides were eluted with a linear gradient from 5 to 50% buffer B over 186 min followed by 80% B wash for 10 min and aqueous re-equilibration at a flow rate of 300 nL/min with a total run time of 230 min. A Q Exactive quadrupole-orbitrap hybrid mass spectrometer (Thermo Scientific, Bremen, Germany) was used in DDA mode. Survey full-scan MS spectra (m/z 350–1800) were acquired with a resolution of 70,000. Source ionization parameters were as follows: spray voltage, 2.5 kV; capillary temperature, 300 °C; and s-lens level, 44.0. The MS peak width at half height was < 30 s. The MS/MS spectra of the 12 most intense ions from the MS1 scan with a charge state ≥ 2 were acquired with the following options: resolution, 17,500; isolation width, 2.0 m/z; normalized collision energy, 27%; ion selection threshold, 4.00E ± 03 counts; and peptide match, ‘preferred’.

### Secretome analysis

#### Construction of MMP-2-suppressed cells and stable isotopic labeling with amino acids in cell culture (SILAC)

A stable HCT116 cell line with suppressed MMP-2 expression was constructed by transfecting a pGIPZ lentiviral vector harboring an shRNA sequence for *MMP2*. The shRNA was designed as described^64^. Another shRNA with a scrambled sequence was designed for use as a negative control. Transfection with the indicated shRNA vector was performed using the X-tremeGENE™ HP DNA transfection reagent (Roche, Basel, Switzerland) according to manufacturer’s instructions. For antibiotic selection, the transfected cells were grown in RPMI 1640 media with 6 µg/mL puromycin (Sigma-Aldrich). After construction of the stable cell lines, decreased expression of *MMP2* gene was confirmed by quantitative RT-PCR.

The established cells were cultured in SILAC RPMI media (RPMI-1640 media deficient in L-lysine and L-arginine; Thermo Scientific, San Jose, CA) supplemented with 10% dialyzed fetal bovine serum (FBS) (dialyzed by ultrafiltration, Sigma-Aldrich), 1 × penicillin/streptomycin (Gibco, Rockville, MD), 253.7 µg/mL L-Arg and 55.2 µg/mL L-Lys. For preparation of heavy SILAC medium, [L-^13^C_6_, ^15^N_4_]-Arg and [L-^13^C_6_, ^15^N_2_]-Lys (Arg10 and Lys8, respectively; Cambridge isotope) were used instead of the conventional amino acids (Arg0; Lys0) used for light SILAC. Cells were grown for at least six doublings. SILAC amino acids were incorporated into more than 95% of the cellular proteome as determined by mass spectrometry (MS). Excess amount of arginine in media may get metabolized by cells to proline leading to reduced quantitative accuracy. This metabolic conversion can be largely prevented by the addition of *L*-proline to the SILAC culture medium^65^. However, in this study, we didn’t add proline in advance and arginine-to-proline conversion was found to be 11.51%.

#### Collection of conditioned media

Grown cells were transferred to an appropriate serum free SILAC medium. After a 24-h incubation, the conditioned medium was harvested, cleared of floating cells and debris by centrifugation (400 g, 10 min, 4 °C) followed by filtration (0.22 µm, Millipore, MA). After addition of 50 mM EDTA and 1 mM PMSF to prevent proteolytic degradation, the conditioned medium was concentrated by ultrafiltration using Amicon Ultra-15 centrifugal filter devices (10 kDa cutoff, Millipore, MA). Buffer was exchanged to 8 M urea, 50 mM Tris (pH 8.2), 75 mM NaCl during the repeated concentration steps. Meanwhile, whole cell lysates were prepared from the cells that remained in the culture dishes. Cells were harvested in the buffer described above and then lysed by sonication. The lysate was centrifuged (12,000 g, 10 min) and the supernatants were collected for further analysis. Protein concentration was determined by a bicinchoninic acid assay (Thermo scientific, Rockford, IL). All protein samples were stored at − 80 °C until use.

#### Tryptic digestion and pre-fractionation

The secretomes (50 µg each) from light and heavy SILAC conditions were pooled. The pooled sample was reduced with 10 mM dithiothreitol at 37 °C for 25 min and alkylated with 15 mM iodoacetamide at 25 °C for 30 min. Then, the sample was diluted by eightfold to decrease urea concentration to at least 1 M and digested with sequencing-grade modified trypsin (Promega, Madison, WI) at 1:25 (w/w) enzyme to protein ratio at 37 °C for 16 h. The tryptic digest was separated based on isoelectric point using an OFFGEL electrophoresis fractionator (Agilent Technology, Santa Clara, CA) as described^59^. The fractionated peptides were collected into 12 fractions, desalted with C_18_ macrospin spin columns (Harvard Apparatus, MA), vacuum-dried (miVac Duo concentrator, Genevac, Suffolk, UK) and stored at − 20 °C until MS analysis.

#### Liquid chromatography and tandem mass spectrometry

LC–MS/MS was performed, as previously described^66^. Peptides were reconstituted in 0.4% acetic acid and analyzed on a reversed-phase C18 column (20 cm × 75 μm i.d., 3 μm, 300 Å, packed in-house; Dr. Maisch GmbH) using an Eksigent MDLC system at a flow rate of 300 nL/min. Before use, the column was equilibrated with 95% mobile phase A (0.1% formic acid in H_2_O) and 5% mobile phase B (0.1% formic acid in ACN). The peptides were eluted with a linear gradient from 5–40%B over 90 min followed by an 80%B wash and re-equilibration with 5%B at a flow rate of 300 nL/min with a total run time of 120 min. The HPLC system was connected to an LTQ Orbitrap XL mass spectrometer (Thermo Scientific, Bremen, Germany) operated in DDA mode. Survey full-scan MS spectra (m/z 300–2000) were acquired in the Orbitrap with a resolution of 100,000. Source ionization parameters were as follows: spray voltage, 1.9 kV; capillary temperature, 275 °C. The MS/MS spectra of the 10 most intense ions from the MS1 scan, with a charge state ≥ 2, were acquired in the ion-trap using the following parameters: isolation width, 2.0 m/z; normalized collision energy, 35%; dynamic exclusion, 30 s.

### Protein identification and quantitation

Raw data files were processed using MaxQuant (ver.1.5.8.2) software with the Andromeda search engine^67^. The MS/MS spectra were searched against the UniProt human database (released in Jan. 2016). With respect to the secretome, sequences for 199 experimentally validated FBS proteins (common Repository of FBS proteins) were added to the human database to reduce false-positives originating from FBS contamination^66,68^. The following search parameters were used: full tryptic specificity allowing N-terminal cleavage to proline, up to two missed cleavage sites, carbamidomethylation of cysteine residues set as a fixed modification, methionine oxidation and N-terminal protein acetylation as variable modifications. The false discovery rate (FDR) was set to 0.01 for proteins and peptides and was determined by searching a reverse database. Maximal allowed precursor mass deviation for peptide identification was 4.5 ppm and maximal fragment mass deviation was 20 ppm. For quantification of SILAC-labeled secretome, multiplicity was set as two to match the number of SILAC labels used (i.e. light and heavy) with Arg10 + Lys8 set as heavy. Relative abundance was measured based on the ratio of MS1 resolved peak areas of the isotopic clusters for the SILAC pairs. For quantification of plasma proteome, label-free quantification (LFQ) was performed with a minimum ratio count of two^69^.

The MS raw files are available via PRIDE^70^ partner repository with the dataset identifier PXD018304.

### Bioinformatics

Ingenuity Pathway Analysis (IPA, Qiagen, http://www.ingenuity.com) was used to determine knowledgebase-oriented protein networks and to generate a heat map presenting the functional annotation related to disease. Perseus (version 1.5.8.4)^71^ was used for all other bioinformatic analyses performed in this study.

We utilized the colon tissue proteomic data generated and published by the Clinical Proteomic Tumor Analysis Consortium (NCI/NIH) (https://cptac-data-portal.georgetown.edu/cptac/s/S037). In this Clinical Proteomic Tumor Analysis Consortium (CPTAC) Cancer proteome confirmatory colon study^50^, 104 colon tumor samples were analyzed by two proteome characterization centers, Vanderbilt University and Pacific Northwest National Laboratory. One hundred tumors were investigated by label-free global proteomic profiling and 95 tumors were analyzed by TMT10-plex labeling quantification. We used the latter TMT10-plex data because the TMT platform usually provides greater precision in quantification than the label-free platform^50^. Other data such as clinical information were also provided by the same CPTAC data portal.

Gene set enrichment analysis (GSEA) was performed for pathway enrichment analysis of CPTAC CRC tissue data^72^. Tumor samples grouped according to the expression level of MMP-2; Top 50% (n = 47), MMP-2 high group and bottom 50% (n = 48), MMP-2 low group. KEGG pathway was used for enrichment analysis and the statistical significance was evaluated based on the multiple testing correction with the false discovery rate < 0.25. The heat map was generated using the Morpheus^73^.

For hierarchical clustering and generation of heat maps of plasma proteome and tissue proteome, we used the built-in tool in Perseus (version 1.5.8.4)^71^. All quantified proteins were Z-scored normalized across the stage and clustered based on Euclidean distance. The number of clusters for plasma proteome (11 clusters) and tissue proteome (16 clusters) were defined based on distance threshold for 1.578.

For scoring activities of FAK-associated transcriptional factors, gene expression data of the same CPTAC CRC cohort from which the tissue proteome was downloaded from the NCI-GDC portal (https://portal.gdc.cancer.gov/) using the TCGA biolinks package (version 2.17.1)^74^. As per standard practice, gene expression data were log2-transformed after 1 was added to each gene expression value. Transcriptional interaction data between five transcription factors (TFs) activated by FAK (FAK-TFs: ETS1, JUN, KLF8, NFκB1, and YBX1)^26^ and their target genes were acquired from the TF-target interaction database available in OmniPath^28^, excluding data derived from computational prediction. A target gene enrichment score for each of the FAK-TFs was calculated by using single sample Gene Set Enrichment Analysis^75^ on gene expression data and presented as a heat map using the ComplexHeatmap package (version 3.11)^76^.

### Western blot analysis

Equal amounts of protein from each sample were separated on SDS-PAGE gels and transferred to PVDF membranes (Bio-Rad, Hercules, CA). The membranes were blocked with 5% skim milk in TBST buffer (20 mM Tris, 137 mM NaCl, and 0.1% Tween 20, pH 7.4) for 30 min at RT and incubated with the appropriate primary antibody overnight at 4 °C. After several washes with TBST, the membranes were incubated with the corresponding IgG-HRP secondary antibodies at a dilution of 1:10,000 for 1 h at RT, washed, and visualized with the ECL chemiluminescent substrate (Bio-Rad, Hercules, CA). Unedited, expanded western blots provided in Supplementary Information. Several western blots were cut prior to antibody hybridization for reagent conservation.

### In vitro cell proliferation, migration assays

Cell proliferation of the constructed cell lines was evaluated using the MTT (3-(4,5-Dimethylthiazol-2-yl)-2,5-Diphenyltetrazolium Bromide)-based colorimetric assay (Invitrogen, V13154), according to the manufacturer instructions. Cells were seeded in 96-well plates at 5 × 10^3^ cells per well and absorbance was measured using a microplate reader at 540 nm in every 24 h for 4 days.

For measuring migration ability of cells, cells were seeded into Oris™ Cell Migration Assay 96-well plates (Tissue Culture Treated) fitted with stoppers (Platypus Technologies, CMA1.101) at a concentration of 5 × 10^5^ cells per well. The cells were then incubated for 4 h in a humidity chamber at 37 °C and 5% CO_2_. After removal of the stopper, the cells were incubated for 16 h. Live cells were stained with 0.5 µg/mL of calcein AM solution and incubated at 37 °C for 1 h. Fluorescence images were obtained using a low light microscope system and the amount of fluorescence was determined on a BioTek Synergy™ microplate reader using 485 and 528 nm excitation/emission filters at a sensitivity of 55 nm.

### Zymography

The proteolytic activity of MMP-2 was measured and visualized using SDS-PAGE zymography, in which 0.1% (w/v) porcine gelatin was incorporated into a 10% polyacrylamide gel. Conditioned medium containing 5 µg protein (concentrated by ultrafiltration if required) was loaded onto the gel and separated by electrophoresis. The gel was washed in distilled water, incubated for 1 h at RT with 2.5% (v/v) Triton X-100 in 50 mM Tris–Cl (pH 7.5), washed again in distilled water and incubated for 16 h at 37 °C with incubation buffer (50 mM Tris–Cl pH 7.5, 10 mM CaCl_2_, 0.01% NaN_3_, 1 µM ZnCl_2_). After a brief wash with distilled water, the gel was stained with Coomassie Brilliant Blue. Unstained white bands of digested substrate in the blue background indicated metalloproteinase activity.

## Supplementary Information

Supplementary Information 1.

Supplementary Information 2.
