# Supplementary material for: Multi-layered proteogenomic analysis unravels cancer metastasis directed by MMP-2 and focal adhesion kinase signaling
Source: Sci Rep. 2021 Aug 24;11:17130. doi: 10.1038/s41598-021-96635-7 (PMC8385024; doi:10.1038/s41598-021-96635-7)
Supplement: Supplementary file 2 — Supplementary Information 2. [file 41598_2021_96635_MOESM2_ESM.pdf]

Supplementary Information

Multi-layered proteogenomic analysis unravels cancer metastasis directed by MMP-2 and focal adhesion kinase signaling

Yumi Kwon[1,2], Seong-Jun Park[1,$], Binh Thanh Nguyen[3,4], Mi Jeong Kim[1], Sejin Oh[5,6], Hwanho Lee[6,7], Narae Park[1,8], Hyun Seok Kim[5,6], Min-Jung Kang[3,4], Byung Soh Min[9], Jin-Won Lee[2], Eun Gyeong Yang[*,1] and Cheolju Lee[*,1,4,8]

[1]Center for Theragnosis, Korea Institute of Science and Technology (KIST), Seoul 02792, Korea
[2]Department of Life Science and Research Institute for Natural Sciences, Hanyang University, Seoul 04763, Korea
[3]Molecular Recognition Research Center, KIST, Seoul 02792, Korea
[4]Division of Bio-Medical Science & Technology, KIST School, Korea University of Science and Technology, Seoul 02792, Korea
[5]Severance Biomedical Science Institute, Yonsei University College of Medicine, Seoul 06229, Korea
[6]Brain Korea 21 PLUS Project for Medical Science, Yonsei University College of Medicine, Seoul 06229, Korea
[7]Department of Systems Biology, College of Life Science and Biotechnology, Yonsei University, Seoul 06229, Korea
[8]KHU-KIST Department of Converging Science and Technology, Kyung Hee University, Seoul 02447, Korea
[9]Department of Surgery, Yonsei University of College of Medicine, Seoul 06229, Korea
[$]Present address: RetiMark Co. Ltd., Seoul 02792, Korea

[*] Corresponding authors

Dr. Cheolju Lee: Center for Theragnosis, Korea Institute of Science and Technology, 5 Hwarangro-14-gil, Seongbuk-gu, Seoul 02792, Republic of Korea; Tel: +82-2-958-6788; Email: clee270@kist.re.kr

Dr. Eun Gyeong Yang: Center for Theragnosis, Korea Institute of Science and Technology, 5 Hwarangro-14-gil, Seongbuk-gu, Seoul 02792, Republic of Korea; Tel: +82-2-958-5031; Email: eunyang@kist.re.kr

Table of contents

Legends of supplemental tables S-3

Legends of supplemental figures S-4

Supplemental figures S-5

Supplemental tables (Table S3−S6) S-31

Legends of supplemental tables

Table S1 The list of identified and quantified proteins in plasma proteome experiment.

Table S2 The list of identified and quantified proteins in secretome SILAC experiments

Table S3 Gene ontology enrichment analysis using Fisher's exact test (Benjamini-Hochberg false discovery rate value <0.02) was performed for the proteins differentially expressed in SILAC (fold-change >1.5, t-test p-value <0.05). All quantified proteins were used as the background list.

Table S4 Summary of hierarchical clustering of plasma proteome and comparison with secretome.

Table S5 Clinical data of samples for tissue profiling data used in this study

Table S6 Summary of hierarchical clustering of tissue proteome and comparison with secretome

Legends of supplemental figures

Figure S1 (A) Schematic workflow of plasma proteome analysis. Depleted plasma proteins from CRC patients at TNM stage 1, 2, 3, and 4 were analyzed. The numbers in the Venn diagram represent those of identified proteins. (B) Information of plasma samples used for profiling data.

Figure S2 Most-significant gene network of proteins identified in the plasma cluster 6 (Enrichment score=41). Network was generated using the IPA tools and node (gene) and edge (gene relationship) symbols were described in the bottom part of the figure. Genes in uncolored notes were not identified the plasma cluster 6 in our data set and were integrated into the computationally generated networks on the basis of the evidence stored in the IPA knowledge database.

Figure S3 Confirmation of constructed stable cell lines used in this study. (A) The proliferation rate of constructed stable cell line and parental cell line, HCT-116 were measured by MTT assay. Results represent the mean ±SD of four replicates. (B-D) Level of MMP-2 were measured in three different methods. (B) Quantitative RT-PCR, (C) western blot of cell conditioned medium, (D) gelatin zymography.

Figure S4 Experimental design and quality validation of sample preparation for secretome-SILAC analysis. (A) Experimental design of forward and reverse SILAC. (B) Proteins in the cell lysate (c) and secretome (s) were analyzed via Western blot using an anti-tubulin antibody. (C) Heavy isotope incorporation rate were confirmed via LC-MS/MS before merge heavy and light sample.

Figure S5 MS data quality check Histograms of Log2 ratios of abundance of quantified secretome. Forward labeled (n=9657) and reverse labeled (n=9651) experiments.

Figure S6 MMP-2 abundances according to stages in CPTAC tissue proteome. Protein abundances correspond to the ratio to internal references provided by CPTAC data. Average MMP-2 abundance from stage II is 1.4-fold higher than stage I ($p \leq 0.05$).

Figure S1

A

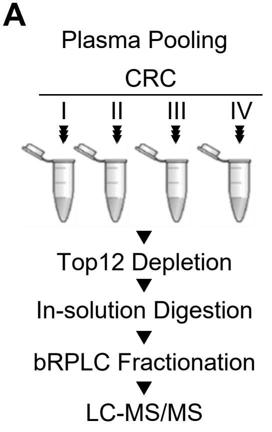

B

	# of samples	ages	sex	
			male	female
Colorectal cancer	187			
stage I	48	63.6±1.0	28	20
stage II	48	63.5±1.2	32	16
stage III	64	62.2±1.0	40	24
stage IV	27	56.4±2.1	17	10

Figure S2

Network generated with the Plasma cluster 6

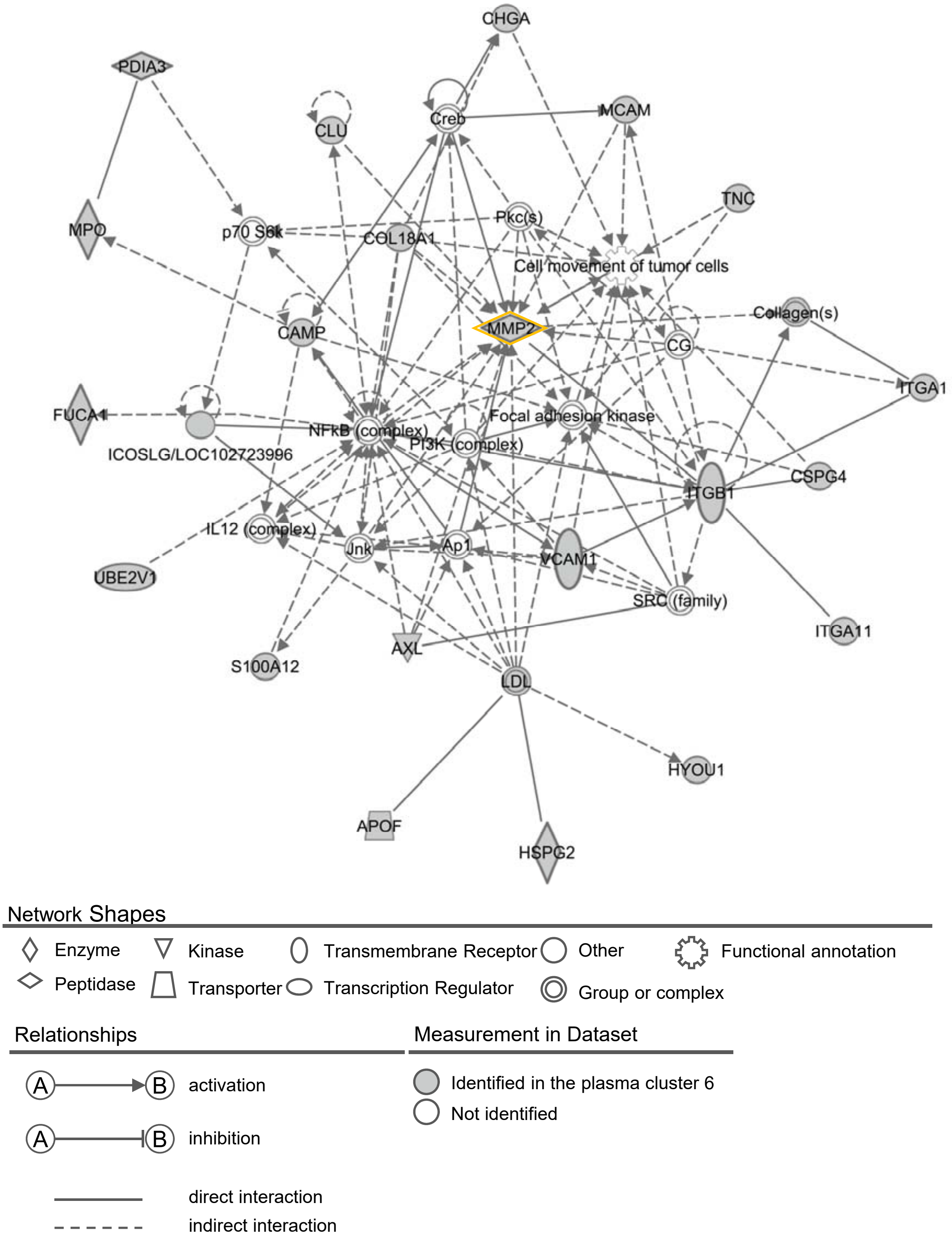

Figure S3

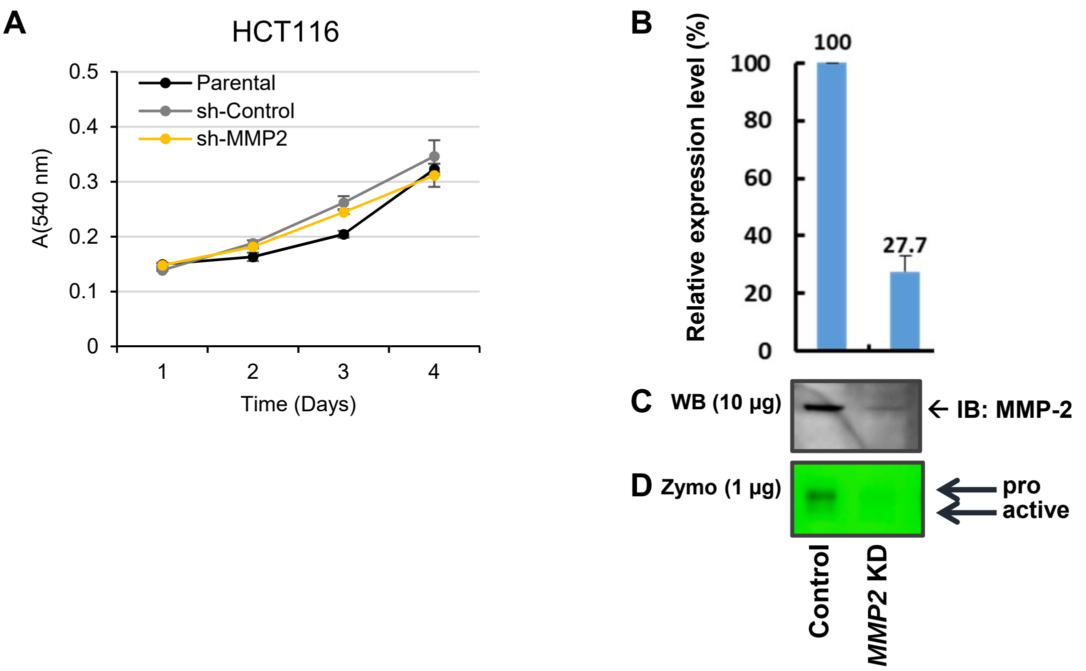
A
HCT116
0.5
0.4
0.3
0.2
0.1
0
A(540 nm)
Parental
sh-Control
sh-MMP2
1
2
3
4
Time (Days)
B
Relative expression level (%)
100
80
60
40
20
0
100
27.7
C
WB (10 µg)
IB: MMP-2
D
Zymo (1 µg)
pro
active
Control
MMP2 KD

Figure S4

A

SILAC experiment	SILAC media	Isotope Composition	Cell line
Forward	Light	Arg0+Lys0	HCT116 shRNA-scramble
	Heavy	Arg10+Lys8	HCT116 shRNA-*MMP2*
Reverse	Light	Arg0+Lys0	HCT116 shRNA-*MMP2*
	Heavy	Arg10+Lys8	HCT116 shRNA-scramble

B

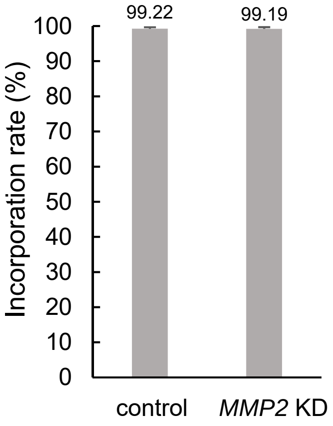

C

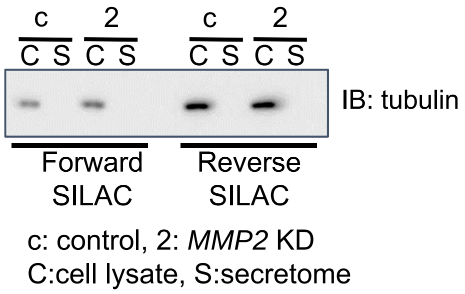

Figure S5

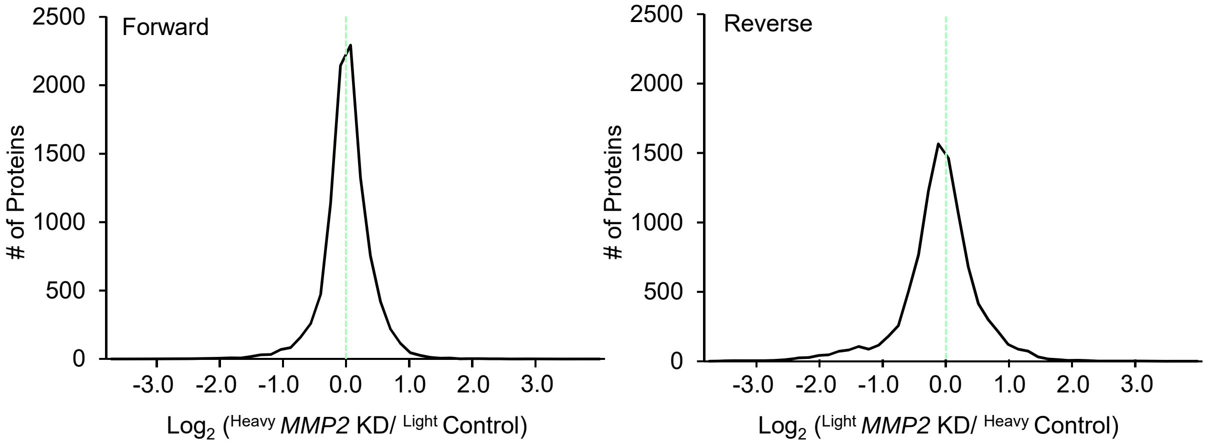
Forward
2500
2000
1500
1000
500
0
# of Proteins
-3.0 -2.0 -1.0 0.0 1.0 2.0 3.0
Log2 (Heavy MMP2 KD/ Light Control)
Reverse
2500
2000
1500
1000
500
0
# of Proteins
-3.0 -2.0 -1.0 0.0 1.0 2.0 3.0
Log2 (Light MMP2 KD/ Heavy Control)

Figure S6

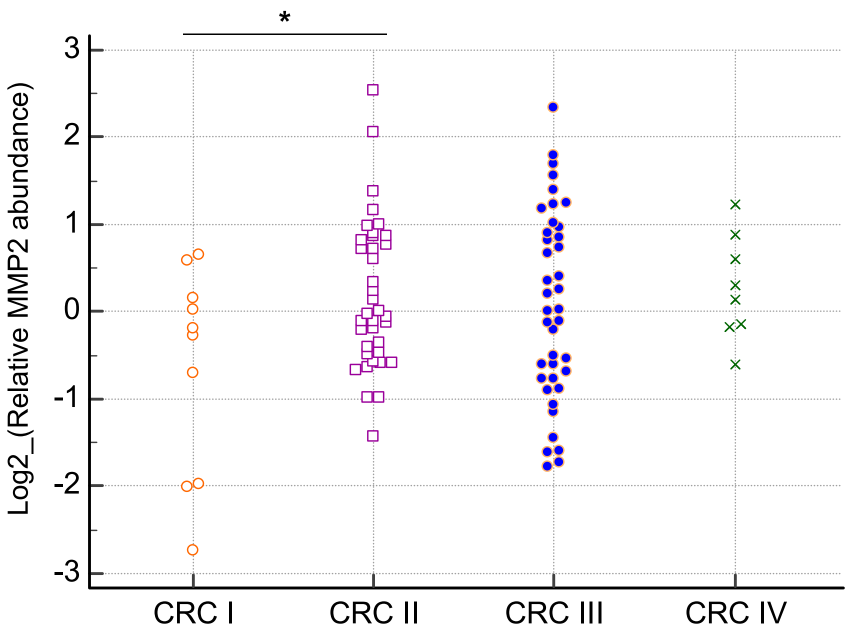

*
3
2
1
0
-1
-2
-3
Log2_(Relative MMP2 abundance)
CRC I
CRC II
CRC III
CRC IV

Original Images

Original image of **Figure 3F-ITGB1** (predicted band size: 130-140 kDa)

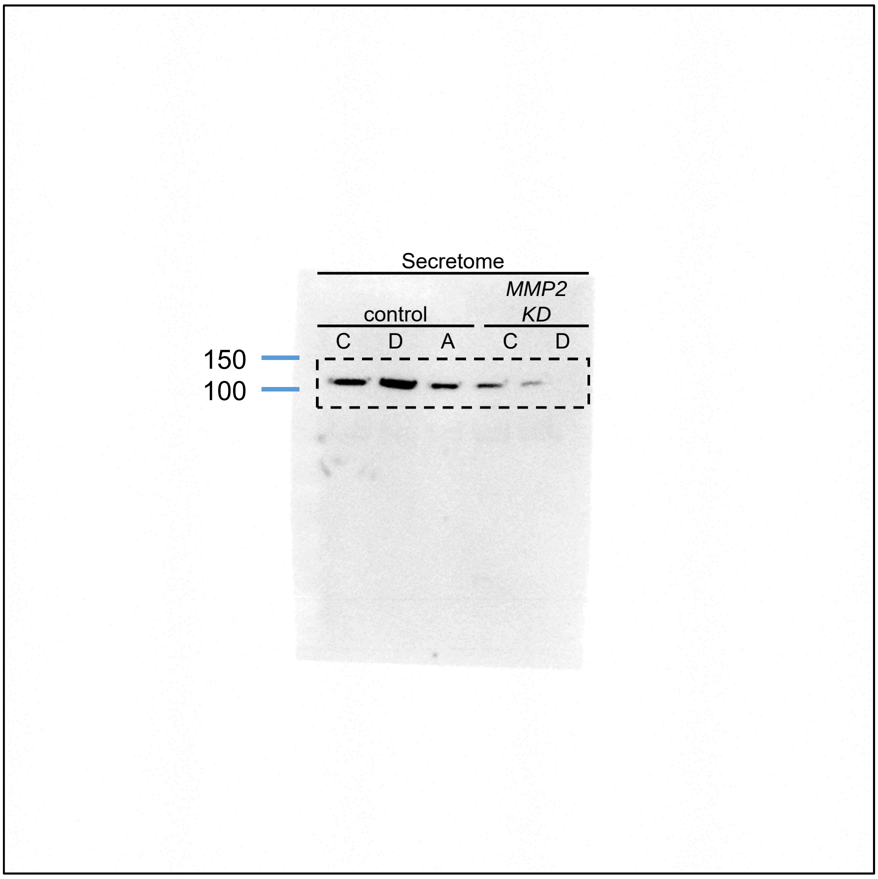

Densitometry

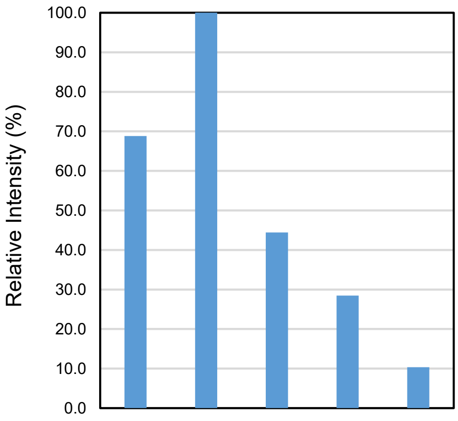

Original image of **Figure 3F-CD9 (24 kDa)**

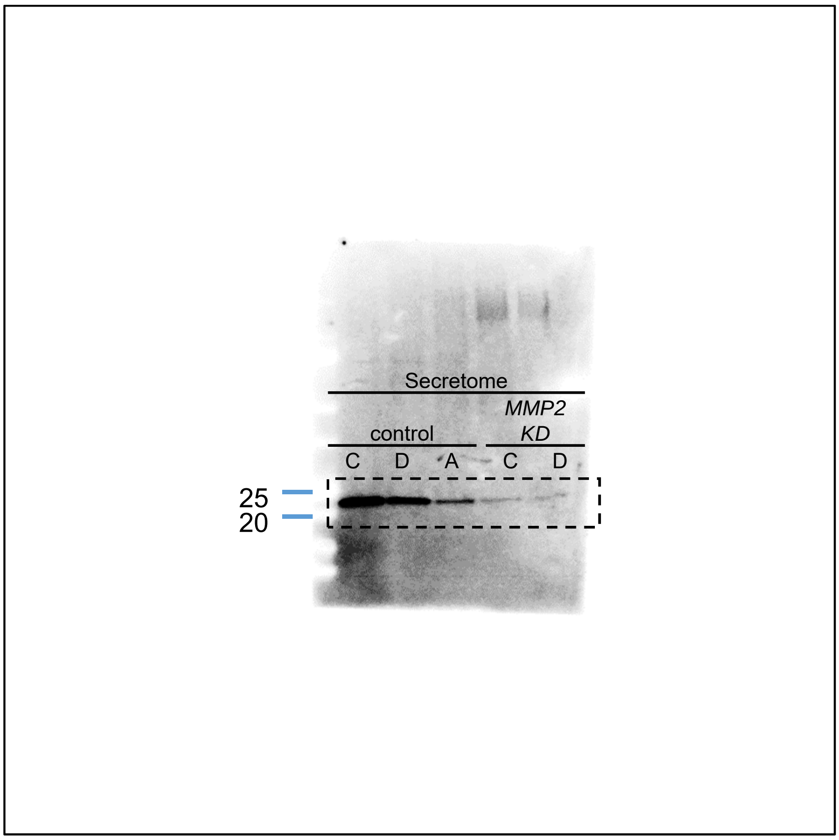

Densitometry

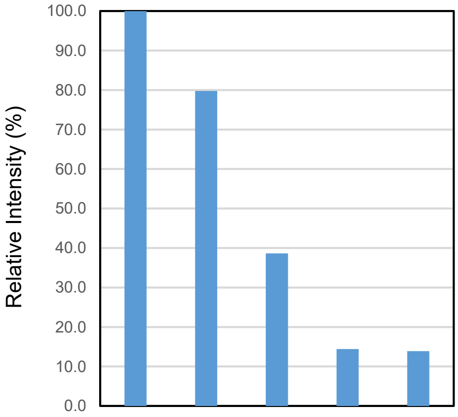

Original image of **Figure 3F-AXL (98 kDa)**

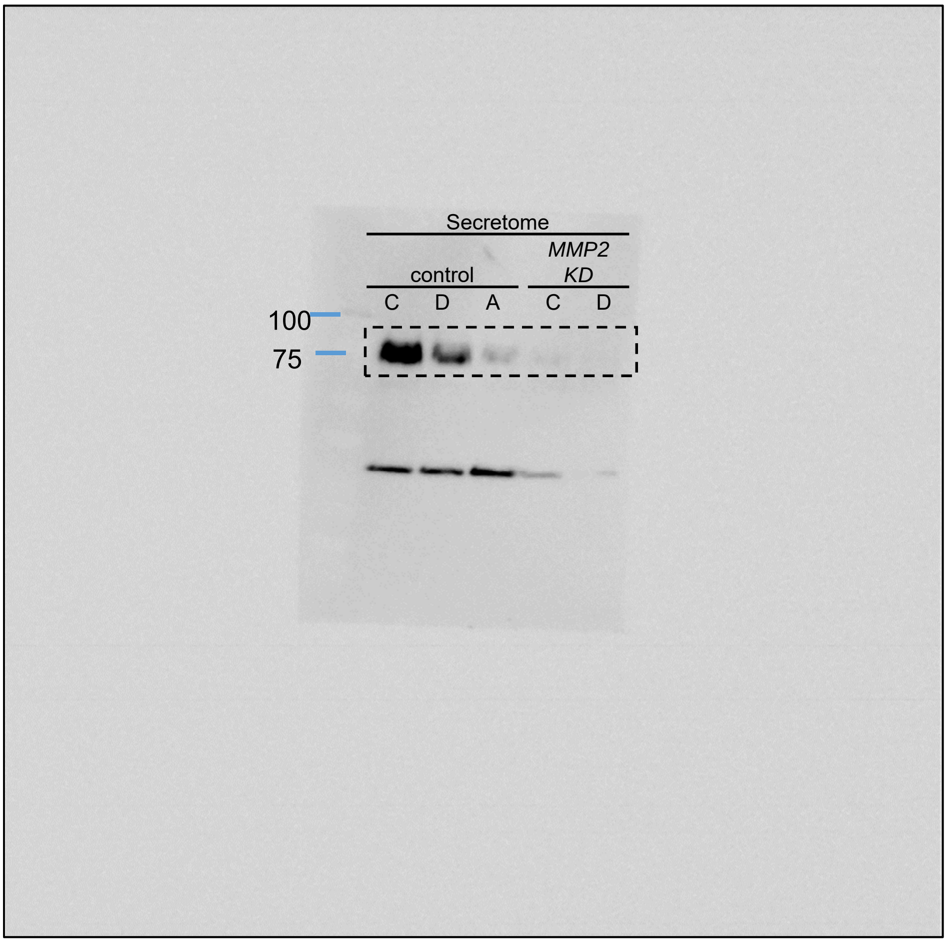

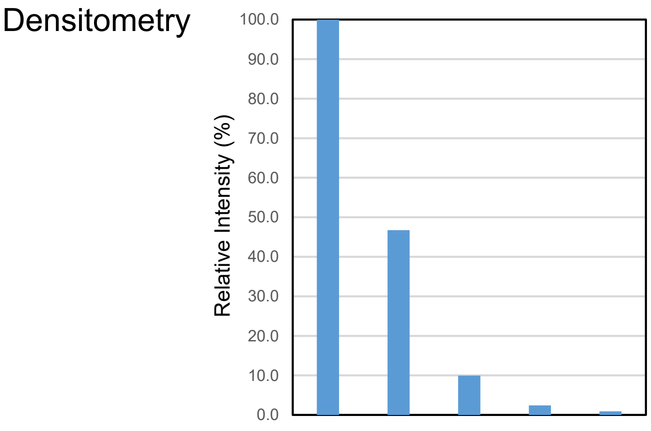

Original images of **Figure 3F-FGFR4** (87.9 kDa)

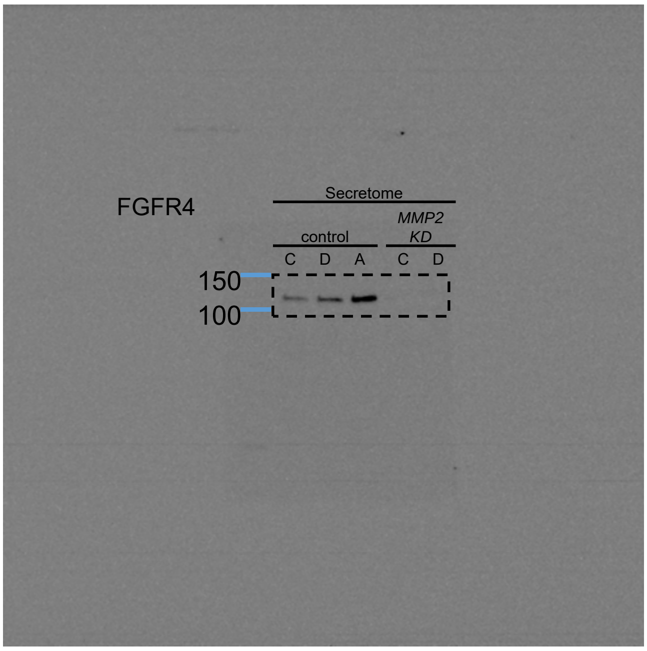

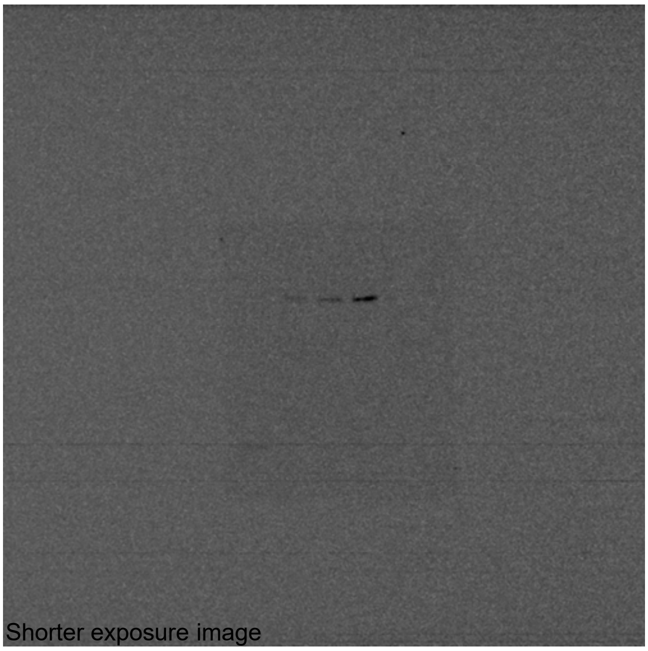

Densitometry

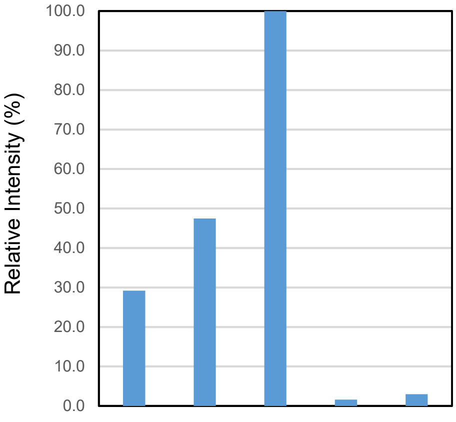

Original images of **Figure 3F-CST3 (13 kDa)**

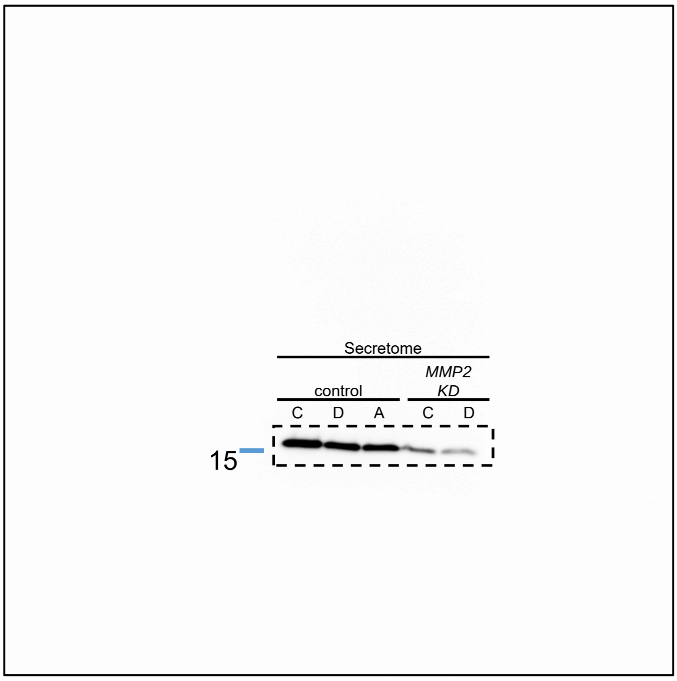

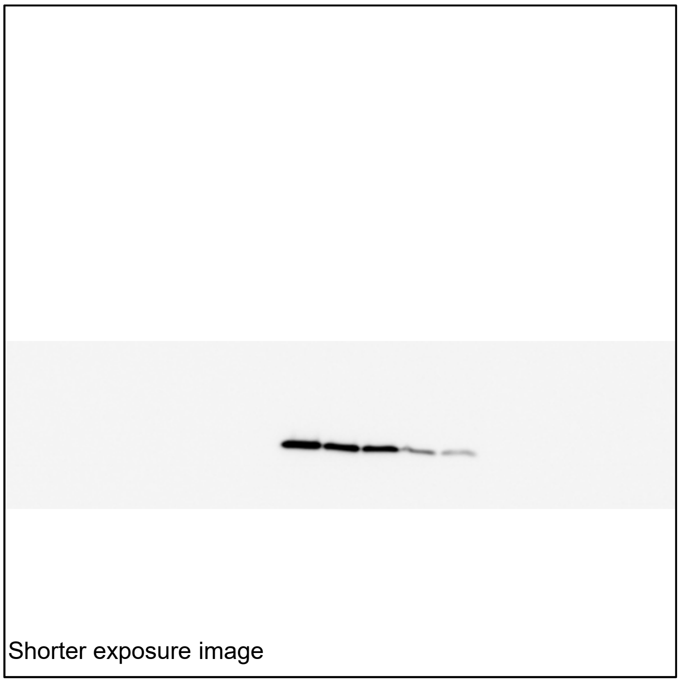

Densitometry

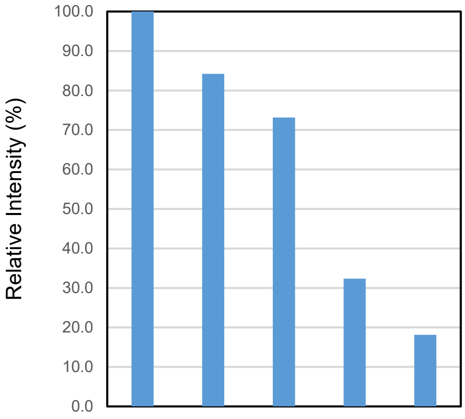

Original images of **Figure 3F-CLU** (52.4 kDa)

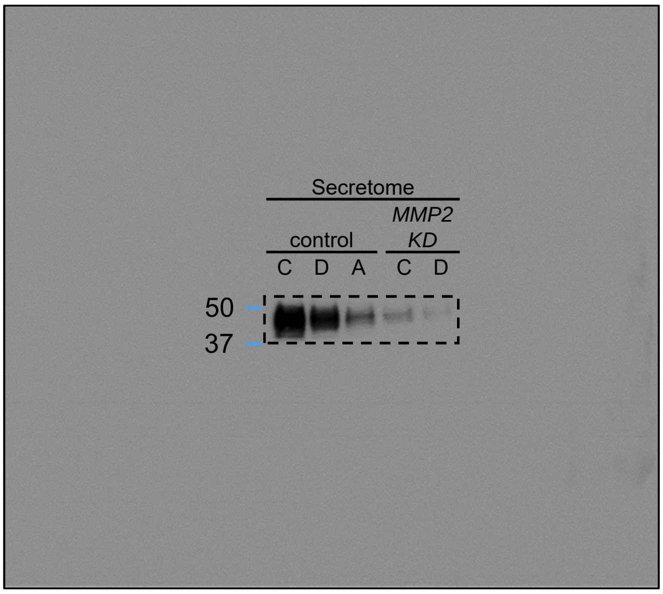

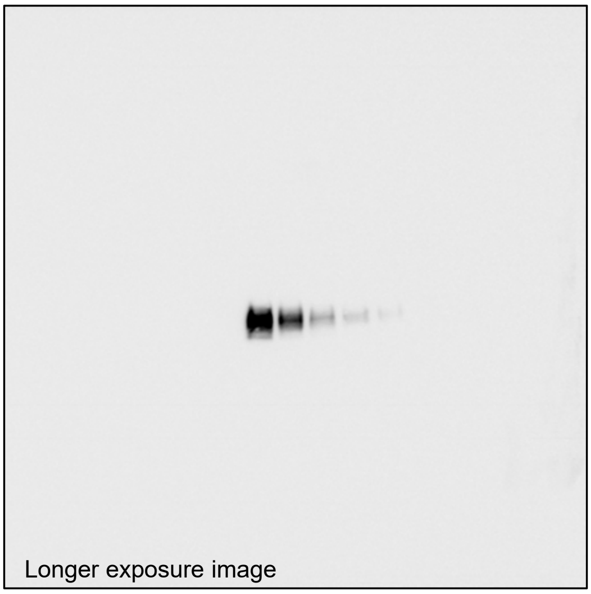

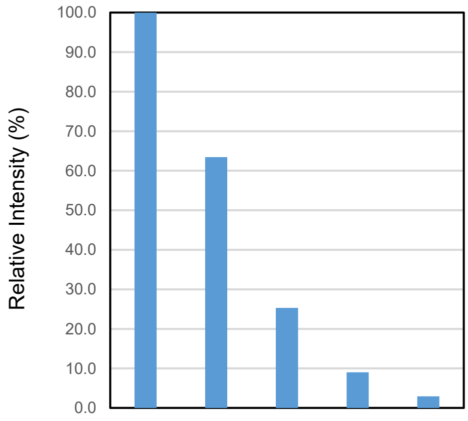

Original image of **Figure 3F-Gelatin Zymography**

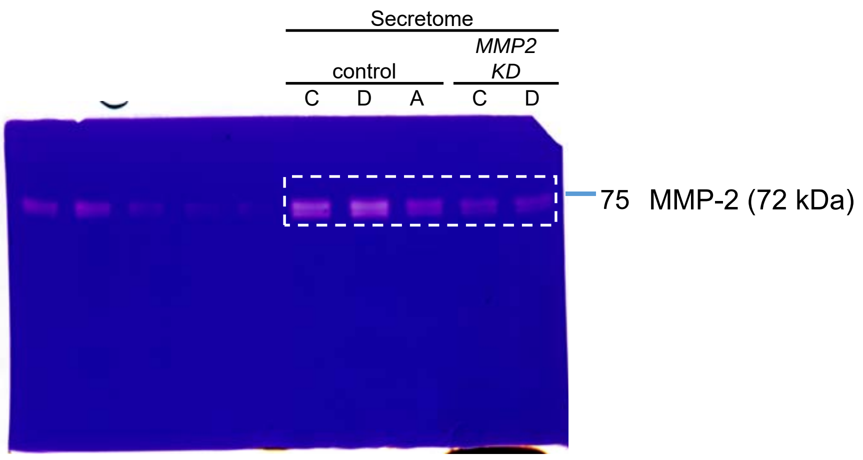

Densitometry

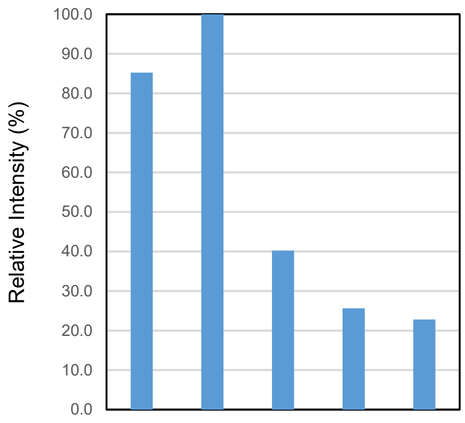

Original image of **Figure 3F-CBB**

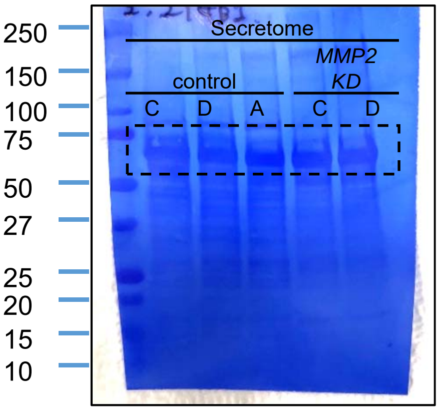

Densitometry

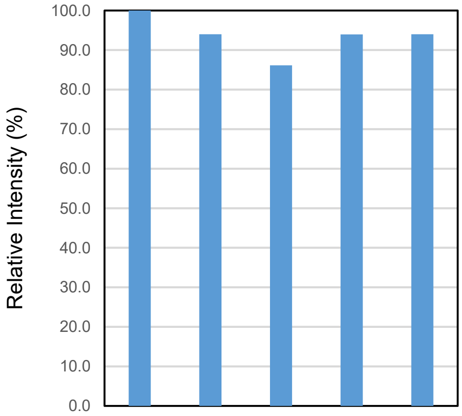

Original images of **Figure 6E-FAK (125 kDa)**

Replicate #1

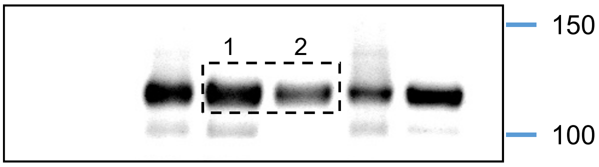

1: Control
2: ARP-100

Replicate #2

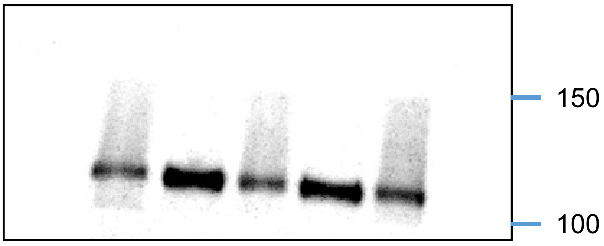

Replicate #3

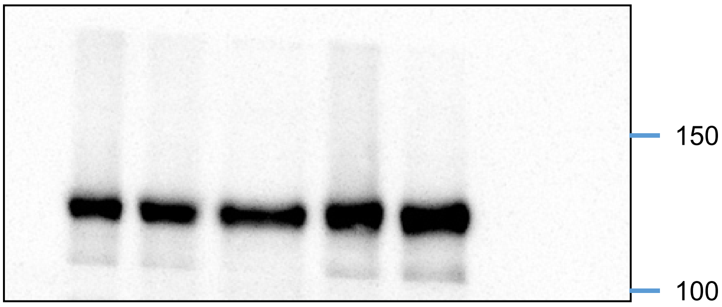

Original images of **Figure 6E-pFAK (Y397, 125 kDa)**

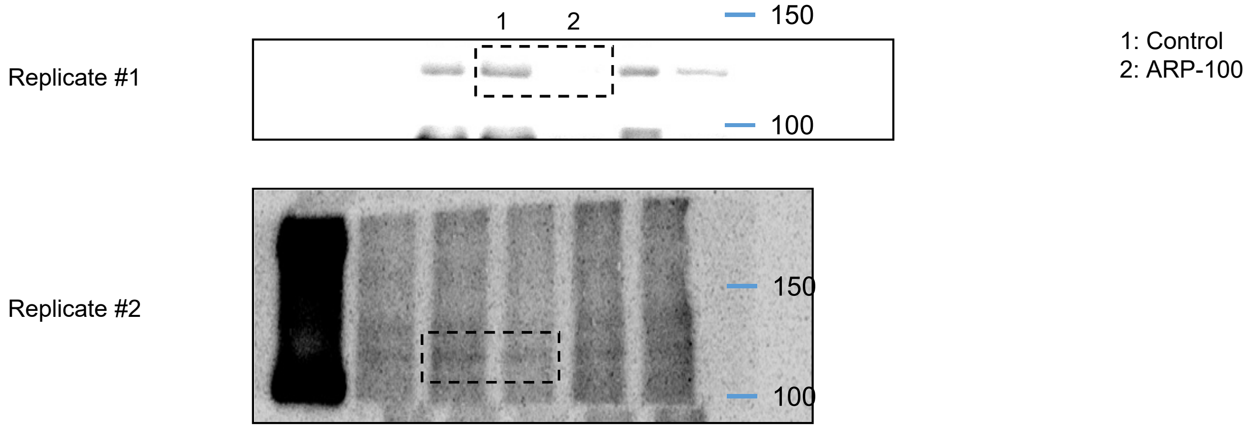

Original images of **Figure PI3K (85 kDa)**

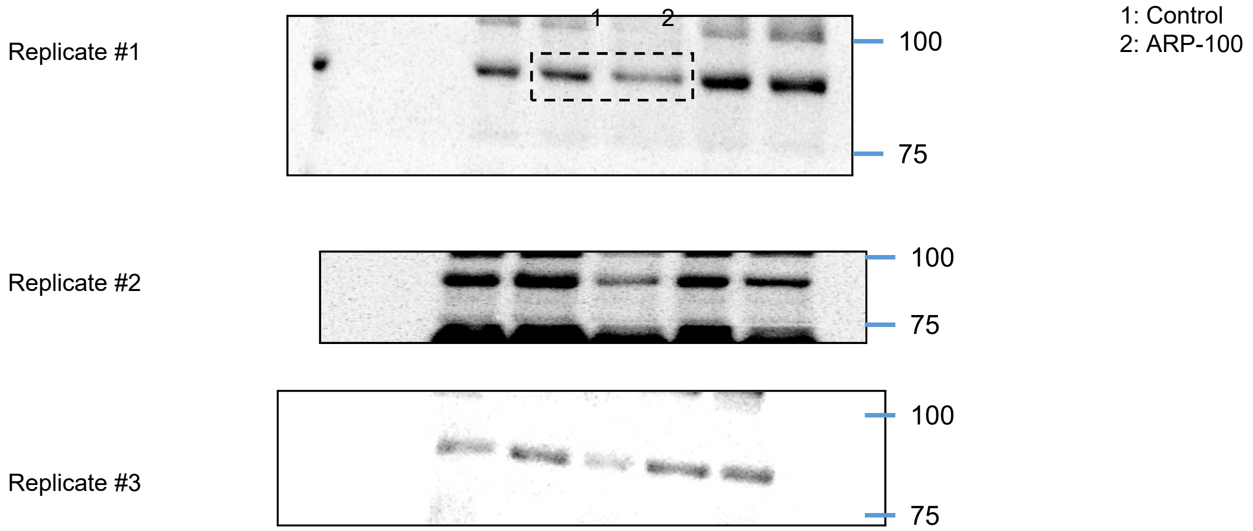

Original images of **Figure 6E-pPI3K (85 kDa)**

Replicate #1

Replicate #2

Replicate #3

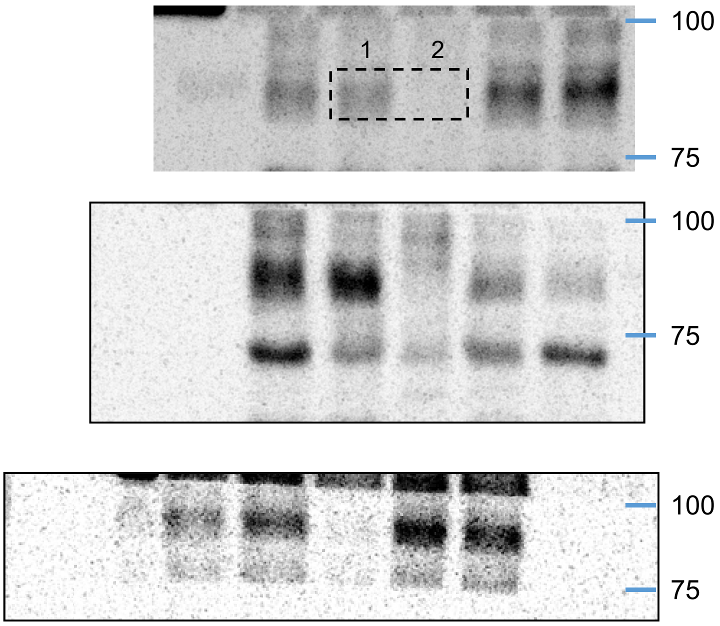

1: Control
2: ARP-100

Original images of **Figure 6E-ERK (44/42 kDa)**

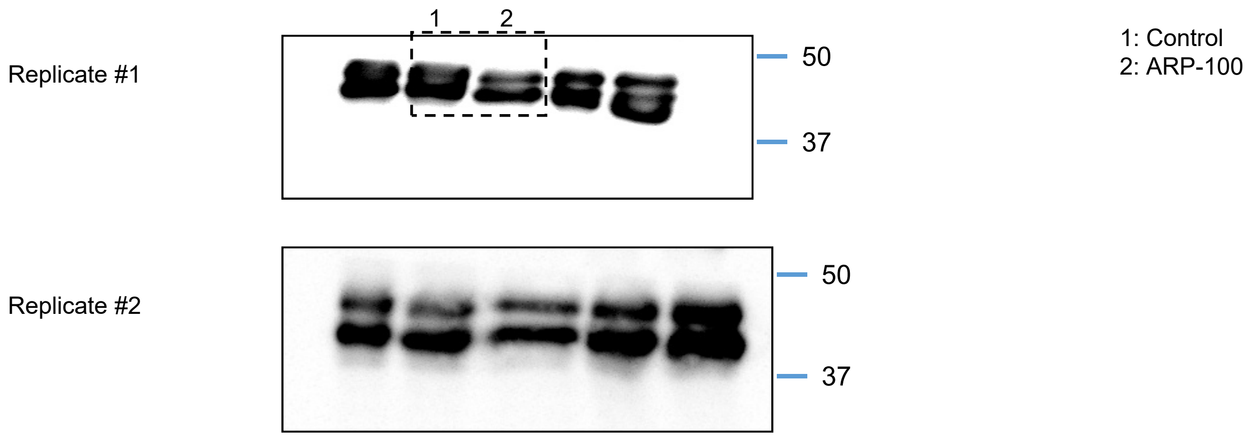

Original images of **Figure 6E-pERK (44/42 kDa)**

1: Control
2: ARP-100

Replicate #1

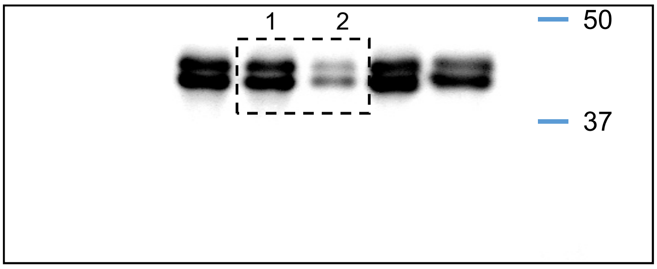

Replicate #2

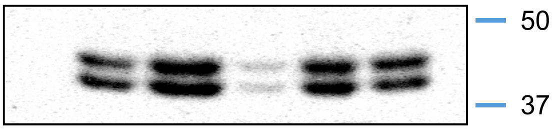

Replicate #3

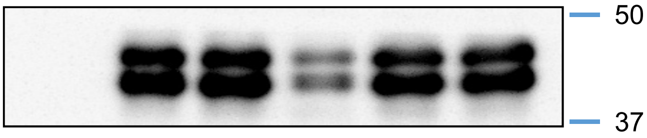

Original images of **Figure 6E-JNK (48 kDa)**

1: Control
2: ARP-100

Replicate #1

Replicate #2

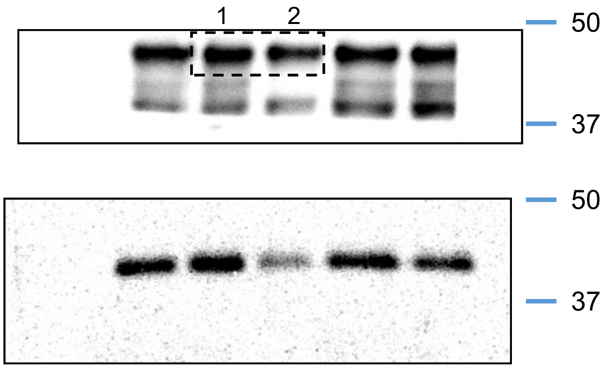

Original images of **Figure 6E-pJNK (48 kDa)**

1: Control
2: ARP-100

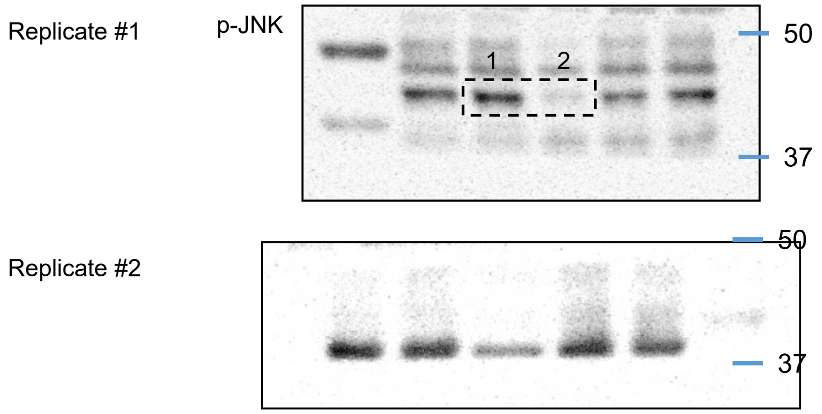

Original images of **Figure 6E-β-actin (42 kDa)**

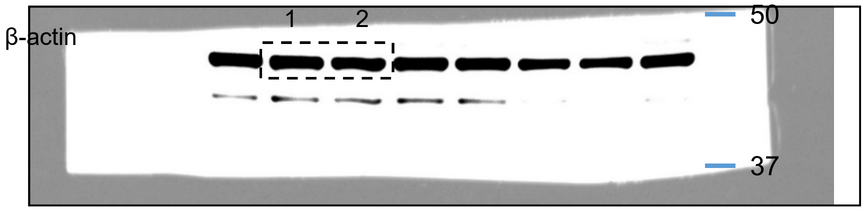

1: Control
2: ARP-100

Original images of **Figure S3B- MMP-2 (72 kDa)**

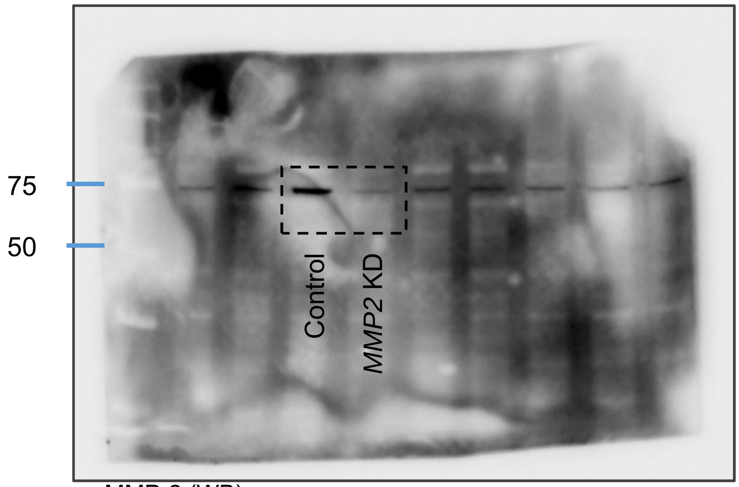

MMP-2 (WB)

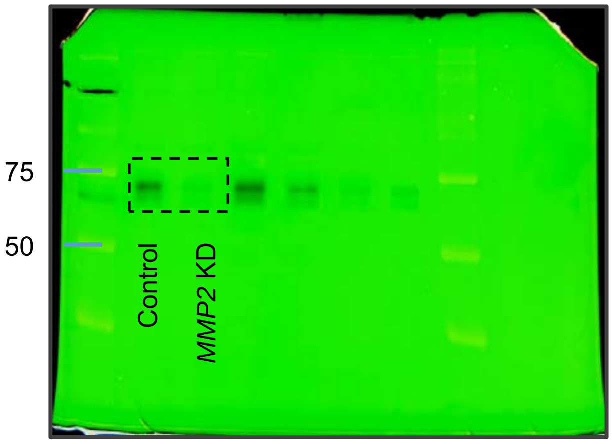

MMP-2 (Zymography)

Original images of **Figure S4-tubulin (50 kDa)**

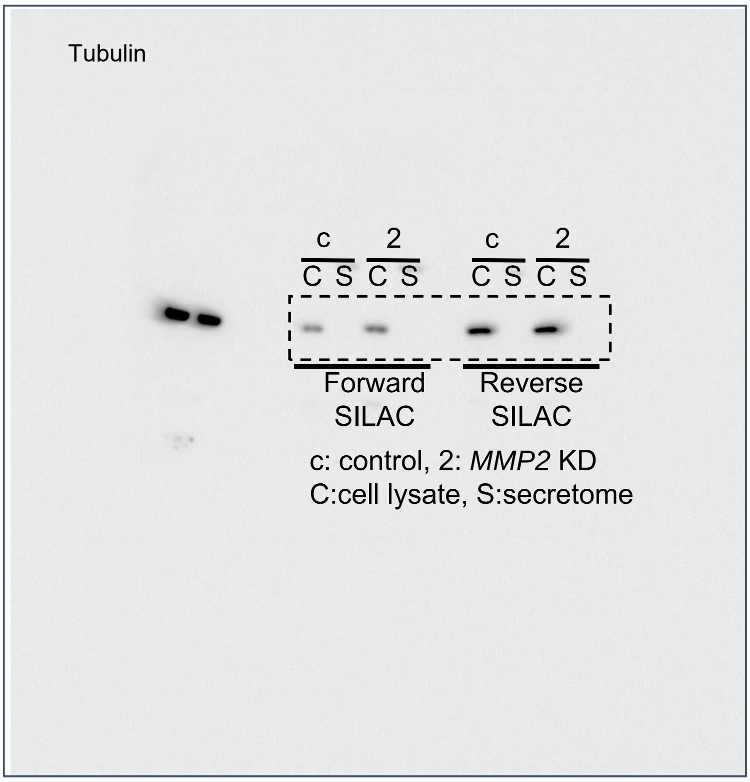

Supplemental table 3. Gene ontology enrichment analysis using Fisher's exact test (Benjamini-Hochberg false discovery rate value <0.02) was performed for the proteins differentially expressed in SILAC (fold-change >1.5, t-test p-value <0.05)

	**Category value**	**Enrichment factor**	**-log(p-value)**	**Associated proteins**
GOMF	Receptor Inhibitor Activity	47.34	4.83	3
	Serine-Type Endopeptidase Inhibitor Activity	14.03	5.59	6
	Peptidase Inhibitor Activity	9.60	5.29	7
	Endopeptidase Inhibitor Activity	9.60	5.29	7
	Glycosaminoglycan Binding	9.18	5.84	8
	Endopeptidase Regulator Activity	8.84	5.04	7
	Polysaccharide Binding	8.28	5.49	8
	Pattern Binding	8.28	5.49	8
	Peptidase Regulator Activity	7.36	4.52	7
	Carbohydrate Binding	6.06	6.57	12
	Receptor Binding	3.46	4.39	13
GOBP	Extracellular Structure Organization	5.49	5.13	10
	Extracellular Matrix Organization	5.49	5.13	10
	Polysaccharide Metabolic Process	4.79	2.94	6
	Cell-Cell Signaling	4.51	3.20	7
	Signaling	4.37	3.12	7
	Cell Communication	4.09	3.67	9
	Cell Motility	3.73	4.05	11
	Locomotion	3.07	4.14	14
	Anatomical Structure Development	2.49	4.55	20
	Response toChemical Stimulus	1.93	3.34	22
	Developmental Process	1.90	4.26	28
	Signal Transduction	1.88	3.51	24
	Response toStimulus	1.55	3.50	34

Supplemental Table 4. Summary of hierarchical clustering of plasma proteome and comparison with secretome.

Plasma cluster	Graphic Trend*	Number of proteins Identified in		
		Plasma	Both secretome and plasma	Decrease more than 1.5 fold in *MMP2* KD secretome
1	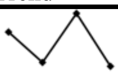	60	27	4
2	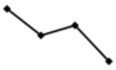	23	11	1
3	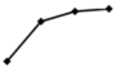	130	76	12
4	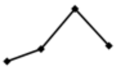	62	31	6
5	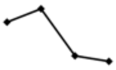	50	24	6
6	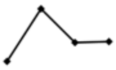	98	56	16
7	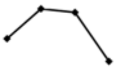	106	50	7
8	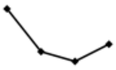	48	17	1
9	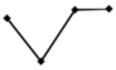	106	50	4
10	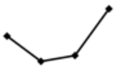	244	92	11
11	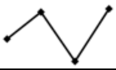	184	89	12

*Each of the 4 points in the graphic trend represents the averaged z-score of the protein expression level of patients at the same CRC stage (I-V) which proteins are associated in the same plasma clusters.

Supplemental Table 5. Clinical data of samples for tissue profiling data used in this study

			sex	
	# of samples	ages	male	female
Colorectal cancer	95			
stage I	10	37.7±6.4	7	3
stage II	37	32.1±5.3	14	23
stage III	40	32.1±5.7	14	26
stage IV	8	32.8±4.5	5	3

Supplemental Table 6. Summary of hierarchical clustering of tissue proteome and comparison with secretome.

Tissue Clulster	Graphic Trend*	Numbers of proteins identified in		
		Tissue	Both tissue proteome and secretome	Decrease more than 1.5 fold in *MMP2* KD secretome
1	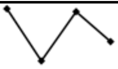	159	79	4
2	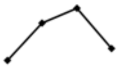	195	94	5
3	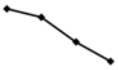	82	35	1
4	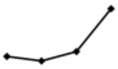	420	214	18
5	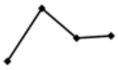	221	103	5
6	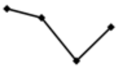	980	408	31
7	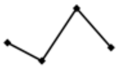	118	51	0
8	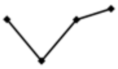	111	46	2
9	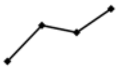	326	157	8
10	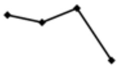	90	41	3
11	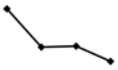	63	30	1
12	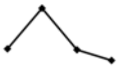	198	108	4
13	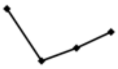	116	67	3
14	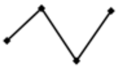	685	360	10
15	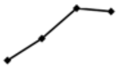	413	199	5
16	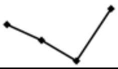	778	413	22

*Each of the 4 points in the graphic trend represents the averaged z-score of the protein expression level of patients at the same CRC stage (I-V) which proteins are associated in the same plasma clusters.
